# Medicinal and edible plants in the treatment of dyslipidemia: advances and prospects

**DOI:** 10.1186/s13020-022-00666-9

**Published:** 2022-09-29

**Authors:** Ying Hu, Xingjuan Chen, Mu Hu, Dongwei Zhang, Shuo Yuan, Ping Li, Ling Feng

**Affiliations:** 1 China Academy of Chinese Medical Sciences Guang’anmen Hospital, Beijing, 100053 China; 2 China Academy of Chinese Medical Sciences, Beijing, 100700 China; 3 Beijing University of Chinese Medicine, Beijing, 100029 China; 4 The First Affiliated Hospital of Zhengzhou University, Zhengzhou, 450000 China; 5 Henan Key Laboratory of Children’s Genetics and Metabolic Diseases, Children’s Hospital Affiliated to Zhengzhou University, Henan Children’s Hospital, Zhengzhou Children’s Hospital, Zhengzhou, 450018 China; 6 Beijing University of Chinese Medicine Third Affiliated Hospital, Beijing, 100029 China

**Keywords:** Medicinal and edible plants, Dyslipidemia, Lipid-lowering, Signaling pathway

## Abstract

Dyslipidemia is an independent risk factor of cardiovascular diseases (CVDs), which lead to the high mortality, disability, and medical expenses in the worldwide. Based on the previous researches, the improvement of dyslipidemia could efficiently prevent the occurrence and progress of cardiovascular diseases. Medicinal and edible plants (MEPs) are the characteristics of Chinese medicine, and could be employed for the disease treatment and health care mostly due to their homology of medicine and food. Compared to the lipid-lowering drugs with many adverse effects, such as rhabdomyolysis and impaired liver function, MEPs exhibit the great potential in the treatment of dyslipidemia with high efficiency, good tolerance and commercial value. In this review, we would like to introduce 20 kinds of MEPs with lipid-lowering effect in the following aspects, including the source, function, active component, target and underlying mechanism, which may provide inspiration for the development of new prescription, functional food and complementary therapy for dyslipidemia.

## Introduction

Dyslipidemia is the metabolic disorder of plasma lipids and lipoproteins [1], and the overall prevalence rate of dyslipidemia among Chinese residents over 40 years old was 43% according to the latest cardiovascular health and disease report [2]. Dyslipidemia is considered as an independent risk factor of CVDs [3], which globally lead to high mortality, disability, and medical expenses [4, 5]. In addition, studies have revealed that the high non-high-density lipoprotein cholesterol was the main reason for ischemic heart disease and stroke, leading to approximately 3.9 million deaths throughout the world [6]. Since primary prevention plays a crucial role in decreasing the incidence of CVDs [7, 8], advances in modifying dyslipidemia are of great help to reduce morbidity and mortality associated with CVDs [4, 9].

Currently, as the first-line lipid-lowering drugs, statins have adverse reactions such as myalgia, liver damage, and diabetes, especially used in large doses [10–12]. Similar side effects such as myopathy, liver enzyme elevations, and cholelithiasis are also found in the progress of dyslipidemia therapy when the patients treated with fibrates [4, 13]. Although the targeted therapeutic drugs proprotein convertase subtilisin/kexin type 9 (PCSK9) inhibitors have been developed, the high cost and unverified safety limit further clinical applications [4, 13]. In addition, unsatisfactory therapeutic effect and drug resistance were also found in some patients [14]. Obviously, the development of additional and alternative treatments is still highly necessary for dyslipidemia therapy.

Over the past 5000 years, traditional Chinese medicine (TCM) has been used to prevent and treat various diseases. Based on the abundant clinical experiences and researches, the efficacy and safety of TCM have been verified. For example, TCM has shown remarkably potential in the fight against worldwide pandemic disease COVID-19, cancer and cardiovascular diseases [15–17]. The theory of medicine and food homology originated from ancient times and developed for thousands of years in China [18]. MEPs come from nature, and could be employed for disease treatment and health care [19]. These plants have unique pharmacological characteristics and chemical structures, and the containing bioactive components are extraordinary sources of drug discovery [20–22]. Simultaneously, these substances can be made into various diets or food stuff, which are widely consumed in daily life [23]. Compared to the lipid-lowering drugs with many adverse effects, MEPs exhibit great advantages such as high efficiency, good tolerance and commercial value. In recent years, although reviews on the treatment of dyslipidemia with TCM formulas, natural products and dietary supplements have been reported [24–28], the review of herbal medicines with food properties is still rare. In this article, we will introduce 20 kinds of MEPs commonly used in clinic and daily life with lipid-lowering effects in the aspects of source, efficacy, target and underlying mechanism, which may provide inspiration for the development of new drugs, functional foods and complementary therapy.

## Methodology and strategy

This review focused on experimental studies in vivo and in vitro. Primarily, MEPs were selected with reference to the Catalogue of Food and Chinese Medicine Homologous substances issued by the National Health Commission of China [29, 30], *Interpretation of Food and Chinese Medicine Homologous Substances* [31], and *Chinese Pharmacopoeia (2020 edition)* [32]. Secondly, according to the reference books and China National Knowledge Infrastructure (CNKI) database, the professional names, common names, and main bioactive components of the plants were collected. Further, publications about the MEPs were searched in PubMed, Web of Science, Google Scholar, and CNKI using relevant medical subject headings (MeSH) and keywords, including the names and active components of the plants, "dyslipidemia", "hyperlipidemia", "cholesterol", "triglyceride", and “lipid metabolism”. Finally, the relevant experimental studies in the past five years (from January 1, 2017 to January 1, 2022) were retrieved.

## MEPs for treating dyslipidemia

**Fig. 1:**
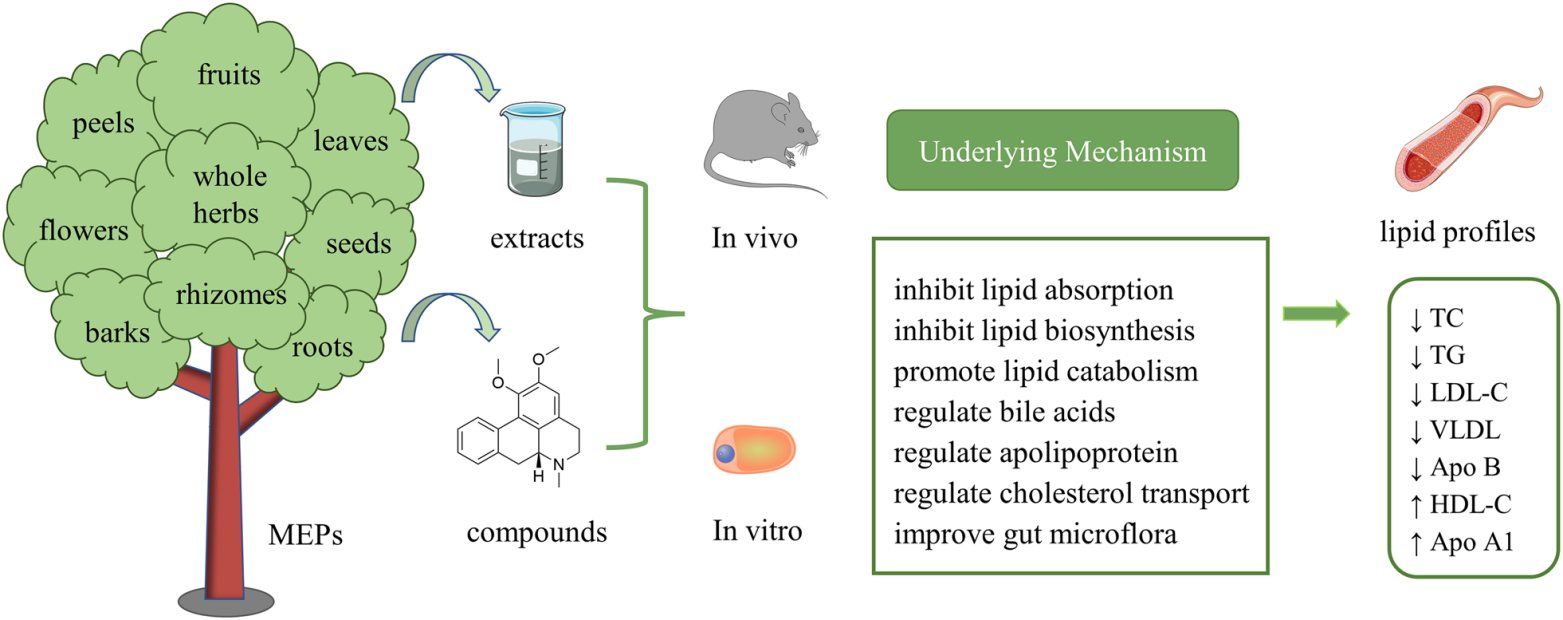
Schematic diagram of the effects and mechanisms of MEPs in modifying dyslipidemia. HDL-C, high-density lipoprotein cholesterol; LDL-C, low-density lipoprotein cholesterol; MEPs, medicinal and edible plants; TC, total cholesterol; TG, triglyceride; VLDL, very low-density lipoprotein

We found that many kinds of MEPs, including the parts of barks, flowers, fruits, leaves, peels, rhizomes, roots, seeds, and the whole herbs, have lipid-lowering effects (Fig. 1). Next, we will introduce these plants in detail.

## Barks

### *Cinnamomi cortex* (Rougui)

*Cinnamomi cortex*, commonly known as cinnamon, is the dried inner bark of *Cinnamomum cassia* Presl of the family Lauraceae [31, 32]. It is one of the essential spices traditionally used to flavor foods in African, Asian and European countries, as well as a folk herbal medicine to treat diseases [33]. There are two main types of cinnamon namely Ceylon cinnamon (*Cinnamon zeylanicum* Blume) and Chinese Cassia (*Cinnamom aromaticum* Ness) [34]. The former grows in Sri Lanka and southern India, and the latter grows in China, Indonesia and Vietnam [35]. Studies have revealed that cinnamon contains chemical components such as cinnamic acid, linoleic acid, oleic acid, essential oil, eugenol, diisobutyl phthalate, and cinnamaldehyde [36]. These bioactive ingredients endow it with antioxidant, anti-inflammatory, antibacterial, antifungal, anticancer and antidiabetic pharmacological effects [37]. Most importantly, cinnamon and its polyphenolic compounds have therapeutic effects on dyslipidemia.

In hyperlipidemia albino rats, after supplementation of cinnamon powder (4 g/kg body weight) for 30 days, the total cholesterol (TC), triglyceride (TG) and low-density lipoprotein cholesterol (LDL-C) levels in serum significantly decreased, whereas the concentration of high-density lipoprotein cholesterol (HDL-C) elevated [38]. Apart from regulating lipid particles, cinnamon also showed great potential in treating metabolic diseases associated with dyslipidemia. In obese mice treated with a high-fat diet and 1% cinnamon extracts for 14 weeks, the body weight gain as well as the serum TC and TG were significantly reduced compared to the control [39]. Long-term high-fat dietary intake not only leads to dyslipidemia and weight gain, but also causes abnormal blood sugar and insulin resistance. Cinnamon polyphenol (100 mg/kg) administered for 12 weeks can decrease serum lipid profiles and glucose of rats fed with high-fat diet, alleviate inflammatory response and inhibit oxidative stress. The underlying mechanism is associated with the activation of transcription factors and antioxidative defense target genes mediated by sterol regulatory element-binding protein (SREBP) 1c, liver X receptor α (LXRα), peroxisome proliferator-activated receptors α (PPARα), NF-κB and Nrf2 signaling pathway [40].

In addition to Chinese cassia, other varieties of cinnamon have also been found to improve lipid metabolism. In hypercholesterolemia mice model induced by quail yolk, TC content in serum decreased after administration of cinnamon *(Cinnamomum burmannii*) for 28 days [41]. Similarly, cinnamon (*Cinnamomum zeylanicum*) bark extract supplement can reduce the blood levels of TC and TG in hyperlipidemia albino rats induced by Triton X-100 injection [42]. In vitro, cinnamate in Ceylon cinnamon showed inhibitory activity of 3-hydroxy-3-methyl-glutaryl-CoA (HMG-CoA) reductase. Phenolic compounds such as gallic acid, catechin, and epicatechin might decrease cholesterol synthesis and absorption by inhibiting pancreatic lipase, cholesterol esterase, and cholesterol micellization [43].

## Flowers

### *Chrysanthemi flos* (Juhua)

*Chrysanthemi flos* (chrysanthemum) is the dried flower head of *Chrysanthemum morifolium* Ramat. of Compositae [31, 32]. It is famous as a beautiful ornamental plant, which has been used for horticulture, decoration, cut flowers, garland making, and religious ceremonies in many countries [44]. Chrysanthemum originated in China and has traditionally served as flower tea for healthcare and herbal medicine to treat diseases for more than 3000 years [45]. According to different origins and processing methods, it can be classified into four types, including "Boju", "Chuju", "Gongju" and "Hangju" [31]. Many phenolic compounds beneficial to human health have been found in chrysanthemum [46], such as caffeoylquinic acids, phenolic acids and flavonoids [47, 48]. Attributing to these bioactive ingredients, chrysanthemum possesses effects of antioxidation, anti-inflammation, antiobesity, and hypolipidemia [49].

Hangju is one of the most popular high-quality chrysanthemums both for tea and medicine in China. The 0.2% and 0.4% Hangju extract can attenuate the serum lipid concentrations, weight gain and inflammatory response of obese rats induced by a high-fat diet. The underlying mechanism is to activate adenosine monophosphate-activated protein kinase (AMPK) signaling pathway, suppress lipid synthesis gene expression and adipogenesis-related enzyme activity in white adipose tissue and liver, as well as increase gene expression involved in fatty acid oxidation [50]. Another study indicated that Hangju extract (1, 2 or 4 g crude drugs/kg/d) administration for 8 weeks can reduce the levels of TC, TG, LDL-C and LDL/HDL in serum of hyperlipidemia rats, increase serum HDL-C level, and alleviate oxidative damage induced by oxidized LDL (ox-LDL) in vitro [51]. Chrysanthemum has pleasing appearance and phytopharmacological activities attributed to its various flavonoids, such as luteolin, apigenin, acacetin, diosmetin and their glycoside derivatives [47, 52]. These compounds might be the bioactive components for lipid-lowering. Luteolin (50 mg/kg·bw/d) and luteoloside (25 mg/kg·bw/d) in chrysanthemum given for 6 weeks were reported to improve serum lipid profiles of TC, TG, LDL-C and apolipoprotein B (ApoB). It can modulate the enzymes activities of fatty acid β-oxidase (FaβO), cholesterol 7 alpha-hydroxylase (CYP7A1), liver lipase (HL) and diacylglycerol acyltransferase (DGAT) to promote the fatty acid activation and β-oxidation, cholesterol conversion to bile acids and triglyceride metabolism. Meanwhile, fatty acid and cholesterol synthesis were decreased by inhibiting the activities of fatty acid synthase (FAS) and HMG-CoA [53].

## Fruits

### *Citri sarcodactylis fructus* (Foshou)

*Citri sarcodactylis fructus*, commonly known as bergamot, is the dried fruit of *Citrus Medica L. var. Sarcodactylis* Swingle of family Rutaceae [31, 32]. Bergamot is native to Calabria in southern Italy, and is used to treat or cure various symptoms, including fever, sore throat and infectious diseases [54, 55]. In TCM, bergamot can used to alleviate chest or stomach pain, bloating, anorexia, vomiting, cough and excessive phlegm [32]. It can be processed into edible preserved fruits and is widely used in the folks to promote digestion, improve appetite, and resolve phlegm [56]. Bergamot contains flavonoids, coumarins, volatile oil, polysaccharides, amino acids, inorganic elements and other bioactive chemical components [57]. It exerts a high antioxidant and selective antibacterial activity, growth stimulation on gut-beneficial bacteria and protective effect on human microvascular endothelial cells [58]. Notably, evidence has accumulated that bergamot has hypolipemic activity and hepatoprotective effects [59].

Bergamot extract (0.85 and 0.56 mg/ml) was reported to reduce the cholesterol content, lipid droplet accumulation and reactive oxygen species levels in murine pre-adipocytes 3T3-L1 cells [60]. The lipid-lowering mechanism is related to the inhibition of hydroxymethyl glutaryl-CoA reductase (HMGCR) and membrane transporters Niemann-Pick C1 Like 1 (NPC1L1), which leading to the reduction of cholesterol biosynthesis and absorption.[61].

Natural bergamot polyphenolic fraction contains more than 40% of flavonoids, such as neoeriocitrin, naringin, neohesperidin and bruteridin [62], which might be the effective components for dyslipidemia and nonalcoholic steatohepatitis. Studies found that bergamot polyphenolic fraction (50 mg/kg/d) administered for 11 weeks can reduce the levels of TG, LDL-C and glucose in blood of mice, alleviate oxidative stress reaction, and improve the key histological and pathophysiological characteristics of nonalcoholic steatohepatitis induced by a high-fat diet and sugar water [63]. In the hyperlipidemic rat model, bergamot polyphenolic fraction (20 mg/kg/d) supplementation for 90 days has been proved to decrease serum TC, TG, LDL-C and fasting plasma glucose whereas increase HDL-C. The underlying mechanism is to regulate the activity of lipid transfer proteins, including acetyl-CoA acetyltransferase (ACAT), lecithin cholesterol acyltransferase (LCAT), and cholesteryl ester transfer protein (CETP) [64]. In addition, cholesterol absorption can be decreased by inhibiting the activity of pancreatic cholesterol ester hydrolase (pCEH) [65].

Naringin, a flavanone-7-O-glycoside contained in bergamot, can reduce the levels of TC, TG and LDL-C by activating AMPK and downregulating the gene expression of SREBP-1 and SREBP-2. Meanwhile, it can decrease and increase the expression of PCSK9 and low-density lipoprotein receptor (LDLR), respectively, to facilitate cholesterol uptake and degradation [66].

### *Crataegi fructus* (Shanzha)

*Crataegi fructus*, also known as hawthorn, is the dried mature fruit of *Crataegus pinnatifida* Bge. var. *Major* N.E.Br. or *C. pinnatifida* Bge. of family Rosaceae [31, 32]. Hawthorn is a red berry that can be made into juices and snacks with sugar or honey, and is recognized to promote digestion. Hawthorn has been used extensively in folk medicine and food production for centuries [67]. It contains flavonoids, organic acids, triterpenoids, polysaccharides and other chemical components [68]. Studies have reported that hawthorn exerts pharmacological effects such as hypolipidemia, lowering blood pressure, hypoglycemia, anti-inflammation, antioxidation, and anti-atherosclerosis [69, 70].

The ethanol extract of hawthorn contains chemical components including chlorogenic acid, hypericin, isoquercitrin, rutin, quercetin, vitexin and apigenin. It can reduce the lipid contents in serum and modulate the perturbed metabolism pathways induced by a high-fat diet in vivo, and inhibit differentiation and TG accumulation in a dose-dependent manner in vitro [71, 72]. Moreover, hawthorn extract and its polysaccharide can lower blood lipids and glucose by inhibiting alpha-glucosidase and pancreatic lipase [73, 74]. In hyperlipidemia mice, freeze-dried hawthorn powder (1, 2.5 g/kg) administration for 12 weeks improved the lipid disorders induced by a high-fat. It can increase the abundance of intestinal flora and restore the composition of intestinal microbes [75]. In addition, supplemented with hawthorn concentrated juice (10, 15, 20 ml/kg) for 5 weeks, the contents of TC, TG, LDL-C, and very low-density lipoprotein cholesterol (VLDL-C) in serum decreased, while the HDL-C level increased. The improvement of LCAT activity and oxidative stress response may be involved in the regulation of lipid metabolism [76].

Haw pectin penta-oligogalacturonide (300 mg/kg) purified from hawthorn pectin hydrolysates can downregulate the mRNA and protein expression of farnesoid X receptor (FXR) and increase CYP7A1 and apical sodium-dependent bile acid transporter (ASBT) in the small intestine of mice, thereby inhibiting intestinal bile acids reabsorption, promoting hepatic bile acids biosynthesis, and improving cholesterol metabolism [77]. Hawthorn crude glycoprotein has good lipid-lowering effects and antioxidant activities. It can reduce TC and TG levels, and increase HDL-C content [78]. Vitexin, a flavonoid extracted from hawthorn, can decrease serum lipid profiles, blood glucose and adipogenesis by activating AMPKα and inhibiting the expression of downstream proteins CCAAT/enhancer binding protein α (C/EBPα) and FAS [79]. Intriguingly, hawthorn can not only ameliorate blood lipid metabolism, but also inhibit the formation of foam cells, resist the inflammatory reaction and regulate gut microbiota, which has potential anti-atherosclerosis effects [80].

### *Gardeniae fructus* (Zhizi)

*Gardeniae fructus*, the dried ripe fruit of evergreen shrub *Gardenia jasminoides* Ellis of family Rubiaceae, is mainly distributed in tropical and subtropical regions of the world. It has been traditionally used as an edible and medicinal substance for centuries in China [81]. Coincidently, another species named *Gardenia resinifera* Roth., mainly grown in the Indian peninsula, Bangladesh and Myanmar, is an excellent crude drug in Indian medicine [82]. *Gardeniae fructus* contains multiple chemical components, such as iridoids, iridoid glycosides, flavonoids, gardenia yellow pigment, triterpenoids, organic acids, and volatile oil. Among them, geniposide, genipin, gardenoside, iridiod and crocin play essential pharmacological active roles [81, 83]. Studies reported that *Gardeniae fructus* can be used for treating diabetes, depression, Alzheimer’s disease and Parkinson’s disease [84–86]. Recently, its potential therapeutic effects on hyperlipidemia, anti-oxidative stress and anti-atherosclerosis have received much attention.

Research showed that after *Gardeniae fructus* extract (25, 50, and 100 mg/kg) administration for 6 weeks, the serum TC, LDL-C, and TG levels of rats decreased in a dose-dependent manner. The potential mechanism is to regulate the mRNA expression of lipogenesis, including SREBP-1c, FAS, stearoyl-CoA desaturase 1 (SCD1), PPARα, and carnitine palmitoyltransferase 1 (CPT1) [87].

Geniposide, a well-known iridoid glycoside isolated from *Gardeniae fructus*, can decrease the serum TC, TG, LDL-C, VLDL and ApoC3 contents whereas increase HDL-C. It can enhance the phosphorylation of AMPK, increase the protein level of PPARα and decrease SREBP-1c. Meanwhile, lipid accumulation and oxidative stress damage due to non-alcohol fatty liver (NAFLD) were ameliorated via Nrf2/AMPK/mTOR signaling pathways [88]. In atherosclerosis mice model induced by high fat/cholesterol diet, geniposide expedited reversal cholesterol transport, motivated bile acid synthesis and excretion, and attenuated atherosclerosis inflammatory injury by modulating FXR-mediated bile acids liver-gut crosstalk and miR-101/MKP-1/p38 signaling pathways [89, 90]. Geniposide can also inhibit the phosphorylation of p38MAPK and AKT to regulate the expression of downstream genes and proteins, thereby decrease cholesterol uptake, promote cholesterol efflux, inhibit the formation of foam cells and alleviate the progress of atherosclerosis [91].

Genipin, the primary metabolite and aglycon of geniposide [92], can promote lipolysis and accelerate liver fatty acid β‐oxidation via upregulating the gene expressions of hormone-sensitive lipase (HSL), adipose triglyceride lipase (ATGL), CPT1A, and PPARα. As a result, the lipid profiles, body weight, fat accumulation, and insulin resistance decreased [93]. Moreover, genipin inhibits lipid metabolic genes and proteins expression of SREBP-1c, FAS, and SCD1 dose-dependently by regulating miR-142a-5p/SREBP-1c axis, which was verified in vitro as well [94].

### *Hippophae fructus* (Shaji)

*Hippophae fructus* (sea buckthorn), the dried ripe fruit of *Hippophae rhamnoides* L. of family Elaeagnaceae, is widely distributed in China, Russia, Mongolia and most parts of Northern Europe. It has been used for food and pharmaceutical purposes in both Europe and Asia for centuries [95, 96]. Currently, sea buckthorn is extensively applied in food, health care, cosmetics, medicines, and many other fields, and has been made into more than 200 kinds of products such as tea, candies, fruit wine, yogurts, seasoning, freeze-dried fruit powder, and toiletries [96]. According to the report, there are 106 nutrients and 74 bioactive compounds in sea buckthorn, including carbohydrates, proteins, unsaturated fatty acids, vitamins, minerals, polysaccharides, sterols, total triterpenic acids, phenolic acids, flavonoids and so on [97]. Among them, flavonoids and sterols are the main pharmaceutically active components in the treatment of dyslipidemia.

Flavonoids in sea buckthorn have shown potential cardiovascular benefits [98], and play an important role in regulating lipid metabolism [99]. Its administration (100, 200, and 400 mg/kg) for 42 days can reduce the contents of TC, TG, and LDL-C in serum of hyperlipidemia mice fed with a high-fat diet, and increase HDL-C levels. Moreover, there is no adverse impact on heart, liver, spleen and kidney [100]. Studies also found that the mRNA expressions of PPARα, LXRα, ATP binding cassette subfamily A member 1 (ABCA1) and CPT1A increased, while SREBP-2 and its target gene LDLR decreased after sea buckthorn flavonoids treatment [101, 102]. The lipid-lowering effects is achieved by promoting the conversion of cholesterol into bile acids, inhibiting cholesterol de novo synthesis, and accelerating fatty acid oxidation.

Isorhamnetin, quercetin, and kaempferol are important flavonoid compounds in sea buckthorn. Isorhamnetin can increase the protein expression of LXRα and CYP7A1 [102]. Kaempferol and kaempferide have been verified to decrease lipid droplets accumulation and TG levels by down-regulating the expression of lipogenesis-related proteins, including SREBP-1, FAS and SCD1. Meanwhile, the expression of two adipogenic transcription factors PPARγ and C/EBPβ were inhibited [103].

**Fig. 2:**
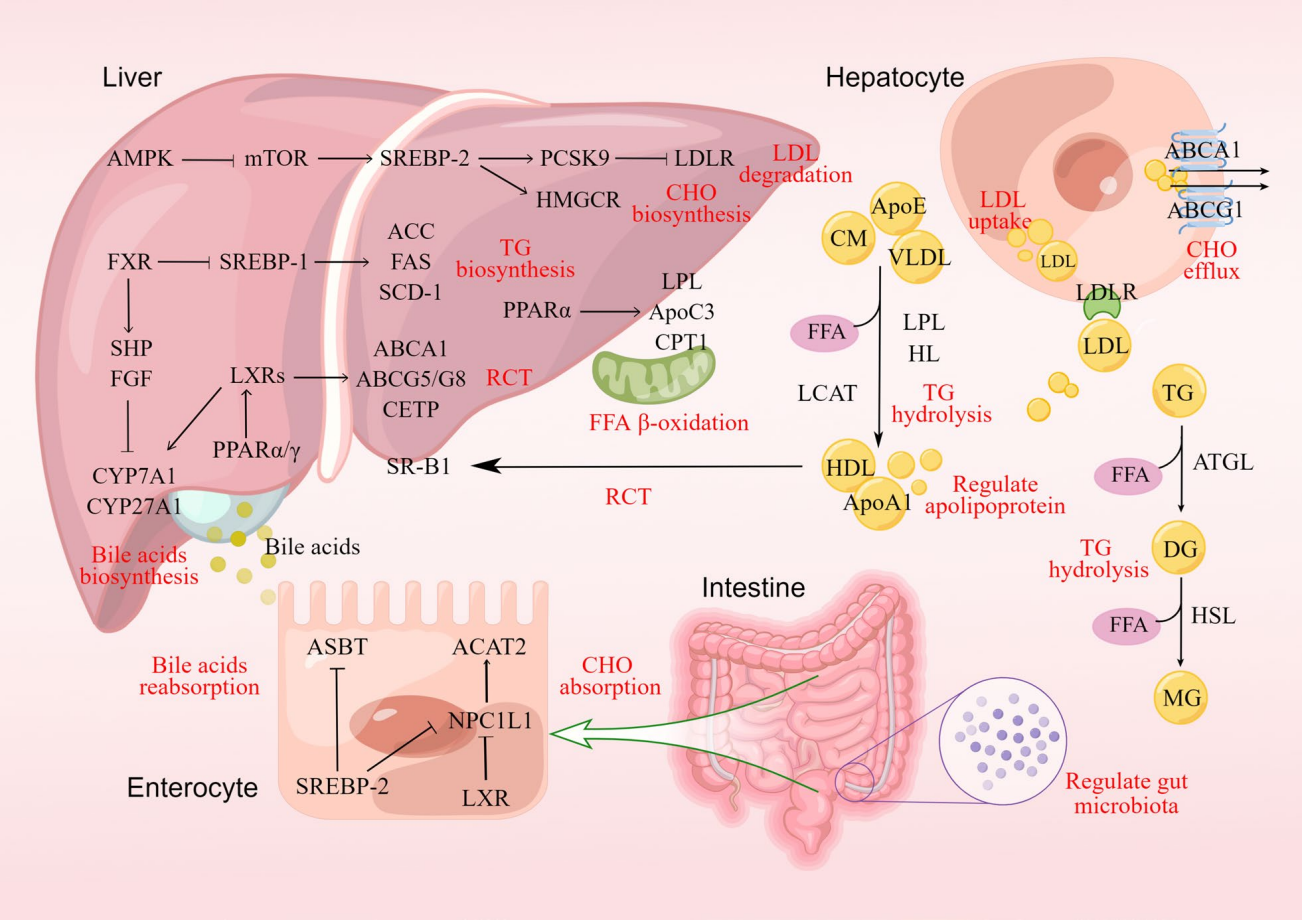
The target genes and proteins of MEPs in the treatment of dyslipidemia, which involves the process of lipid absorption, synthesis, decomposition and metabolism. Red letters indicate the potential lipid-lowering mechanisms. RCT, reverse cholesterol transport. Materials provided by FigDraw (www.figdraw.com)

Sterols (100, 200, and 400 mg/kg) in sea buckthorn, mainly include campesterol, stigmastadienol, sitosterol, stigmastanol and a-Amyrin, were reported to lower the contents of TC, TG, LDL-C and ApoB in blood after treatment for 42 days. It can increase the concentrations of HL, lipoprotein lipase (LPL) and ApoA in serum of hyperlipidemia rats, thus promoting lipid transport, metabolism and decomposition [104]. Figure 2 shows the lipid-lowering target genes as well as upstream and downstream signaling pathways of sea buckthorn and other MEPs.

### *Mori fructus* (Sangshen)

*Mori fructus*, commonly known as mulberry fruit, is the dry ear of *Morus alba* L. of the family Moraceae [31, 32]. It is widely cultivated in Asia, Africa, Europe, North and South America [105], and has been traditionally consumed as medicine and food for a long time [106]. Ripe fresh mulberry fruit is sweet and juicy, which can be eaten raw or processed into juice, jams, preserved fruit and wine [107]. Mulberry fruit is rich in fatty acids, amino acids, vitamins, minerals and other nutrients. It contains phytochemical compounds such as anthocyanins, rutin, quercetin, chlorogenic acid, polysaccharides, polyphenols and alkaloids [108, 109]. Attributing to these bioactive ingredients, mulberry fruit has pharmacological effects including hypolipidemia, hypoglycemia, antioxidation, hepatoprotection, and anti-atherosclerosis [108, 109].

In rats fed with a high cholesterol/cholic acid diet for 4 weeks, lipid profiles in the liver and serum showed an increased tendency. However, the levels of TG, TC and LDL-C in blood of rats administered with mulberry fruit extract (4 g/kg) decreased significantly compared with the control group, while the contents of HDL-C in blood as well as TC, TG and total bile acids in feces increased. These effects were achieved by regulating the expression of mRNA involved in de novo cholesterol biosynthesis, cholesterol efflux, bile acid synthesis and HDL-C formation [110]. In 3T3-L1 cells, incubation with mulberry fruit extract could activate AMPK and inhibit adipogenesis genes, leading to the decrease of intercellular lipid accumulation and TG content [111].

Fresh mulberry fruit is perishable, while freeze-drying mulberry can be stored for a long time and also has effects of improving lipid profiles. It has been reported that lyophilized mulberries can reduce the body weight gain, visceral fat, plasma glucose and TG of hyperlipidemia mice, and at the same time elevate HDL-C contents. Furthermore, the hepatic lipid accumulation, arterial and cardiac wall hypertrophy as well as aortic collagen fiber accumulation attenuated after mulberry fruit (100 and 300 mg/kg) treatment for 3 months [112].

Polysaccharides isolated from mulberry fruit consists of arabinose, galactose, glucose, rhamnose and galacturonic acid. It can improve dyslipidemia, hyperglycemia, oxidative stress and organ damage by regulating gut microbiota [113]. In diabetic rats induced by a high-fat diet and low dose injection of streptozotocin, mulberry fruit polysaccharides (400 mg/kg) treatment for 7 weeks improved lipid profiles, glucose, insulin resistance and hepatic function. Acute toxicity studies revealed that there was no behavioral changes or organic impairment after 1 week administration of mulberry fruit at a dose of 1000 mg/kg [114].

## Leaves

### *Mori folium* (Sangye)

*Mori folium*, commonly known as mulberry leaf, is the dried leaf of *Morus alba* L. of Moraceae [31, 32]. Given its rich bioactive components and nutritional value, mulberry leaf has been widely used in functional foods such as herbal teas, beverages, and noodles in China, Japan, and Korea [115]. Mulberry leaf possesses biological activities of hypolipidemia, hypoglycemia, antibacterial, anti-atherosclerosis, anti-inflammatory and antioxidation [116, 117]. It has been adopted to treat metabolic disorders such as diabetes, obesity, hypertension, dyslipidemia, and fatty liver disease [118]. These beneficial functions are associated with its chemical constituents, including phenols (flavonoids and chlorogenic acid), alkaloids (1-deoxynojirimycin and fagomine), terpenoids and polysaccharides [119, 120].

Studies have reported that mulberry leaf improved dyslipidemia by promoting cholesterol efflux and bile acid synthesis. In hypercholesterolemia rats, mulberry leaf powder (0.9, 0.6, and 0.3 g/kg) treatment for 5 weeks can increase the levels of total bile acids in feces and HDL-C in blood, reduce the contents of TC and LDL-C, and alleviate hepatocyte lipid degeneration. The potential mechanism is to promote cholesterol and total bile acid excretion mediated by FXR and CYP7A1 pathways [121]. Mulberry leaf extract mainly including phenolic compounds such as quercetin and kaempferol, which can lower the levels of TC, TG and LDL-C in serum by enhancing the mRNA expression of CYP7A1, LXRα, ATP binding cassette subfamily G member 5 (ABCG5) and ABCG8, increasing the AMPK activity and suppressing hepatic miR-33 expression [122, 123]. Furthermore, it can decrease the expressions of liver lipogenesis protein SREBP-1, FAS and 1-acylglycerol-3-phosphate o-acyltransferase (AGPAT), whereas increase lipolysis protein contents of CPT1 and PPARα [116].

Chemical compounds isolated from mulberry leaf have shown hypolipidemic effect as well. In the diabetic mice model, flavonoids were reported to improve the levels of TG, TC, LDL-C, HDL-C and glucose in serum, which might be related to the activation of AMPK and CPT1 [124]. Another study demonstrated that mulberry leaf flavonoids and its active metabolite quercetin could reduce excessive cholesterol accumulation both in vivo and in vitro, and play a role in lowering blood lipid via decreasing the mRNA and protein expression of SREBP-2 and HMGCR [125]. Likewise, mulberry leaf phenolic and fiber mixture exhibited lipid-lowering effects via reducing the mRNA and proteins expression of FAS, C/EBR-α and PPARγ as well as regulating the gut microbiota [126]. In ethanol-induced liver injury mouse model, mulberry leaf extract and its chlorogenic derivatives improved lipid profiles, attenuated hepatic inflammation and decreased lipid accumulation [127]. Polysaccharides extracted from mulberry leaf administered for 8 weeks can reduce the TC, TG and LDL-C contents in serum whereas increase the HDL-C levels by inhibiting pancreatic lipase activity [128].

### *Nelumbinis folium* (Heye)

*Nelumbinis folium*, also called lotus leaf, is the dried leaf of *Nelumbo nucifera* Gaertn. of Nymphaeaceae [31, 32]. It is a fragrant Chinese herbal medicine with functions of clearing heat, removing dampness, and raising clearing qi, which has traditionally been used to treat heatstroke, thirst, diarrhea, and fever [129]. Lotus leaf contains alkaloids, flavonoids, polysaccharides, volatile oil and other chemical components, commonly consumed as tea and embraces pharmacological activities of lipid-lowering, anti-obesity, antibacterial and antioxidation [130].

Lipid metabolism disorders and oxidative stress play a key role in the occurrence and development of high-fat diet-induced NAFLD. Lotus leaf powder (600 mg/kg) treated for 18 weeks can modify lipid metabolism disorders, reduce oxidative stress and alleviate NAFLD progress. These effects were achieved by downregulating the mRNA levels of cytochrome P450 2E1 (CYP2E1) and SREBP-1c in liver tissue, as well as inhibiting and enhancing the activity of HMG-CoA and LPL, respectively [131].

Nuciferine, an alkaloid extracted from lotus leaf [132], the main bioactive compounds for dyslipidemia treatment, can regulate the gene expression of key enzymes related to the glycerophospholipid, linoleic acid, and alpha-linolenic acid metabolism pathways in the liver to treat dyslipidemia and NAFLD [133]. Furthermore, it can downregulate the expression of SREBP-1, acetyl-CoA carboxylase (ACC), ATP- citrate lyase (ACLY), and FAS, decrease the levels of TC and TG, and ameliorate liver steatosis [134].

The imbalance of gut microbiota is closely related to the pathogenesis of metabolic diseases, including hyperlipidemia and obesity [135]. Studies have shown that nuciferine can regulate the composition and potential function of intestinal microflora and reduce intestinal permeability, which can prevent weight gain, decrease fat accumulation, and ameliorate lipid metabolism disorders [136, 137]. In vitro, nuciferine can decrease the intracellular TG content and inhibit the proliferation, differentiation, lipid accumulation and adipogenesis of 3T3-L1 preadipocytes. The lipid metabolism related genes PPARγ, SREBP-1, C/EBPα, C/EBPβ, FAS, ACC, and ATGL were involved in this metabolic regulation [138]. Besides, nuciferine contributes to attenuating foam cell formation and atherosclerosis. It can reduce the lipid deposition and TC content of macrophages-derived foam cells via modulating PI3K/AKT/mTOR and PPARγ/LXRα/ABCA1 pathways in a dose- and time-dependent manner [139, 140]. Table 1 summarized the bioactive components, effects, and mechanisms of lotus leaf and other MEPs on dyslipidemia treatment.

**Table 1:** Effects and mechanisms of MEPs in the treatment of dyslipidemia

No	MEPs	Active ingredients	Lipid metabolism	Signaling pathway	Mechanism of action	References
1	*Cinnamomi cortex*	Polyphenol	↓TC, TG, LDL; ↑HDL	1) Inhibit the expressions of SREBP-1c, ACLY, and FAS; 2) Increase the expressions of PPAR-α and IRS	1) Inhibit triglyceride synthesis; 2) Promote fatty acid oxidation; 3) Increase insulin sensitivity	[40]
2	*Chrysanthemi flos*	Extract	↓TC, TG, LDL	1) Downregulate PPAR-γ, SREBP-1c, C/EBP-α, CD36, ACLY, ACC, FAS, SCD1, and DGAT2; 2) Upregulate PPARα and CPT1α; 3) Activate AMPK signaling pathway	1) Inhibit triglyceride biosynthesis; 2) Increase fatty acid oxidation	[50]
		Flavonoids, luteolin, luteoloside	↓TC, TG, LDL, Apo B; ↑Apo A1	1) Inhibit the enzymes activity of FAS, HMG-CoA and DGAT; 2) Increase the activity of FAβO, CYP7A1 and HL	1) Inhibit lipid synthesis; 2) Promote bile acids biosynthesis; 3) Promote fatty acid oxidation	[53]
3	*Citri sarcodactylis fructus*	Extract	↓TC	1) Activate AMPK phosphorylation; 2) Inhibit the activity of HMG-CoA, HMGCR and NPC1L1;	1) Inhibit cholesterol biosynthesis; 2) Inhibit cholesterol absorption	[60, 61]
		Polyphenols	↓TC, TG, LDL, Apo B; ↑HDL, Apo A1	1) Inhibit the activity of pCEH, ACAT and CETP; 2) Enhance the activity of LCAT	1) Inhibit cholesterol esterification and absorption; 2) Improve lipid transport protein system	[64, 65]
		Naringin	↓TC, LDL	1) Upregulate p-AMPKα and LDLR; 2) Downregulate SREBP-1, SREBP-2, and PCSK9	1) Inhibit lipids biosynthesis; 2) Promote cholesterol metabolism	[66]
4	*Crataegi fructus*	Extract; powder; juice	↓TC, TG, LDL; ↑HDL	1) Regulate the perturbed metabolism pathways; 2) Inhibit pancreatic lipase; 3) Increase the LCAT activity	1) Improve gut microflora; 2) Inhibit lipid absorption; 3) Promote reverse cholesterol transport	[71, 72, 75, 76, 80]
		Pectin penta-oligogalacturonide	/	1) Inhibit FXR-FGF15 axis; 2) Increase CYP7A1 and ASBT	1) Promote bile acids biosynthesis; 2) Inhibit intestinal bile acid reabsorption	[77]
		Crude glycoprotein	↓TC, TG; ↑HDL	/	/	[78]
		Vitexin	↓TC, TG	1) Activate AMPKα; 2) Downregulate C/EBPα and FAS	Inhibit de novo lipogenesis	[79]
5	*Gardeniae fructus*	Extract	↓TC, TG, LDL	1) Downregulate SREBP-1c, FAS, SCD1, and PPARγ; 2) Upregulate PPARα and CPT-1	Inhibit biosynthesis of triglyceride and cholesterol	[87]
		Geniposide	↓TC, TG, LDL, VLDL, ApoC3; ↑HDL	1) Enhance the phosphorylation of ACC, AMPKα, and AMPKβ; 2) Upregulate PPARα, LDLR, SR-B1, ABCA1, ABCG1, CYP7A1, CYP27A1, CYP7B1, and CYP8B1; 3) Downregulate SREBP-1c, miR-101 and SR-A; 4) Inhibit FXR-mediated bile acids liver-gut crosstalk	1) Inhibit triglyceride and cholesterol biosynthesis; 2) Decrease free cholesterol esterification and cholesterol uptake; 3) Promote cholesterol efflux; 4) Facilitate reverse cholesterol transport; 5) Motivate bile acid biosynthesis and excretion	[88–91]
		Genipin	↓TC, TG ↑HDL	1) Upregulate the gene expressions of HSL, ATGL, CPT‐1α and PPARα; 2) Downregulate SREBP-1c, FAS, and SCD1	1) Promote triglyceride decomposition; 2) Inhibit triglyceride synthesis	[93, 94]
6	*Hippophae fructus*	Flavonoids; isorhamnetin	↓TG	1) Upregulate PPARα, LXRα, LDLR, CYP7A1, ABCA1 and CPT1A; 2) Downregulate SREBP-2	1) Promote cholesterol metabolism; 2) Inhibit cholesterol de novo synthesis; 3) Accelerate fatty acid oxidation	[101, 102]
		Kaempferol and kaempferide	↓TG	Downregulate SREBP-1, FAS, SCD1, PPARγ and C/EBPβ	Inhibit fatty acid synthesis and adipogenesis	[103]
		Sterols	↓TC, TG, LDL; ↑HDL	1) Increase Apo-A, HL and LPL; 2) Reduce Apo-B	Promote lipids transport and decomposition	[104]
7	*Mori fructus*	Extract	↓TC, TG, LDL; ↑HDL	1) Downregulate miR-33, miR-21, miR-143, FXR, SHP, SREBP-2, PPARγ, and C/EBPα; 2) Upregulate LXR-α, ABCG5, CYP7A1, ABCA1, ApoA-1 and LCAT; 3) Inhibit GPDH activity; 4) Increase AMPK activity	1) Inhibit de novo cholesterol biosynthesis; 2) Increase bile acids synthesis; 3) Promote cholesterol reversal transport; 4) Inhibit adipogenesis and adipocyte differentiation	[110, 111]
		Polysaccharides	↓TC, TG, LDL; ↑HDL	Selective enrich bacteria and reduce intestinal microbial diversity	Regulate gut microbiota	[113]
8	*Mori folium*	Powder	↓TC, LDL; ↑HDL	1) Inhibit FXR expression; 2) Promote CYP7A1 expression; 3) Maintain the ratio of ABCG5/ABCG8	1) Promote cholesterol efflux; 2) Promote bile acids biosynthesis and excretion	[121]
		Extract	↓TC, TG, LDL; ↑HDL	1) Increase CYP7A1, LXRα, ABCG5/ABCG8, CPT1 and PPARα expression; 2) Decrease SREBP-1, FAS, AGPAT and miR-33 expression; 3) Increase the AMPK activity	1) Increase hepatic bile acid biosynthesis; 2) Inhibit lipid biosynthesis; 3) Promote lipid degradation; 4) Promote fecal cholesterol excretion	[116, 122, 123]
		Flavonoids; quercetin	↓TC, TG, LDL; ↑HDL	1) Activate AMPK-PGC-1α signaling pathway; 2) Downregulate SREBP-2, HMGCR, LXRβ and miR-33a; 3) Increase the expressions of CPT-1 and CYP7A1	1) Promote mitochondrial fatty acid oxidation; 2) Improve insulin resistance; 3) Inhibit cholesterol biosynthesis; 4) Promote cholesterol convert to bile acid;	[124, 125]
		Polyphenols and fiber	↓TC, TG, LDL; ↑HDL	1) Downregulate FAS, C/EBP-α and PPARγ; 2) Improve intestinal flora diversity	1) Inhibit triglyceride biosynthesis and adipocyte differentiation; 2) Regulate gut microbiota	[126]
		Polysaccharides	↓TC, TG, LDL; ↑HDL	Inhibit pancreatic lipase activity	Inhibit lipids absorption	[128]
9	*Nelumbinis folium*	Powder	↓TC, TG	1) Downregulate SREBP-1c mRNA; 2) Inhibit HMG-CoA activity; 3) Enhance LPL activity	1) Inhibit synthesis of triglycerides and cholesterol; 2) Promote triglyceride decomposition	[131]
		Nuciferine	↓TC, TG, LDL	1) Downregulate SREBP-1, ACLY, ACC, FAS, PPARγ, C/EBPα and C/EBPβ; 2) Upregulate LCAT activity and PPARγ/LXRα/ABCA1 pathways; 3) Inhibit PI3K/AKT/mTOR pathways; 4) Alter the diversity and composition of gut microbiota	1) Inhibit lipids biosynthesis; 2) Increase cholesterol efflux; 3) Promote autophagy and reduce macrophage foaming; 4) Regulate gut microbiota	[133, 134, 136–140]
10	*Citri reticulatae pericarpium*	Ethanol extract	↓TC, TG, LDL, ApoB; ↑ApoA1	1) Inhibit the activity of HMGCR; 2) Regulate apolipoprotein; 3) Regulate PPARγ-LPL/ATGL/HSL and FXR-HL pathway	1) Decrease cholesterol synthesis; 2) Regulate cholesterol transport; 3) promote triglyceride metabolism	[148, 149]
		Water extract	↓TC, TG, LDL; ↑HDL	Alter the diversity and composition of gut microbiota	Improve gut microflora	[150]
11	*Zanthoxyli pericarpium*	Hydroxy-α-sanshool	↓TC, TG, LDL; ↑HDL	Increase mRNA and protein expression of PPARγ and ApoE	Promote lipid metabolism and lipoprotein transformation	[157]
12	*Dioscoreae rhizoma*	Diosgenin	↓TC, TG; ↑HDL	Activate the catabolic pathway via AMPK	Inhibit cholesterol absorption and facilitate cholesterol excretion	[162]
		Resistant starch	↓TC, TG, LDL; ↑HDL	Increase the relative abundance of probiotics	Regulate gut microbiota	[164]
13	*Polygonati rhizoma*	Extract	↓TC, LDL; ↑HDL	1) Upregulate CPT-1 mRNA expression; 2) Regulate the composition and concentration of amino acids, carbohydrates and esters	1) Promote fatty acid β-oxidation; 2) Regulate endogenous metabolites	[165, 170]
		Polysaccharides	↓TC, TG, LDL; ↑HDL	1) Upregulate PPAR-α and PPAR-β; 2) Downregulate PPAR-γ and SREBP-1c; 3) Regulate gut microbiota and restore the intestinal permeability barrier;	1) Inhibit lipid synthesis; 2) Promote fatty acid oxidation and lipolysis; 3) Regulate gut microbiota	[171, 172]
		Saponin	↓TC, TG, LDL; ↑HDL	Modulate the composition, abundance and diversity of gut microbiota	Regulate gut microbiota	[173]
		Syringaresinol‑di‑O‑β‑D‑glucoside	↓TC, TG, LDL‑C, VLDL‑C, FFA	Promote insulin secretion	Improve insulin sensitivity	[174]
14	*Astragali radix*	Total flavonoids	↓TC, TG, LDL, VLDL; ↑HDL	1) Upregulate FXR, TGR5, CYP7A1, ASBT, AMPKα and CPT1α; 2) Downregulate FAS and SREBP-1c;	1) Promote bile acids synthesis and excretion; 2) Enhance fatty acid oxidation; 3) Inhibit lipid synthesis	[182, 183]
		Astragaloside IV	↓TC, TG, FFA; ↑HDL	1) Activate AMPK, ACC and SREBP-1 phosphorylation; 2) Downregulate SREBP-1, ACC1, FAS and SCD1	Inhibit lipid biosynthesis	[185, 186]
15	*Puerariae radix*	Extract	↓TC; ↑HDL	Activate AMPK and PGC-1α proteins	Promote mitochondrial biogenesis and energy metabolism	[193]
		Puerarin	↓TC, TG, LDL	1) Downregulate SREBP-1c, FAS, SCD1 and HMGCR; 2) Upregulate CPT1, ACOX and HL; 3) Increase the phosphorylation of AMPK and ACC	1) Inhibit triglycerides and cholesterol synthesis; 2) Promote fatty acid oxidation and lipolysis	[194, 195]
		Polysaccharides	↓TC, TG, LDL, FFA; ↑HDL	1) Downregulate SREBP-1 and ACC; 2) Upregulate PPARα and LDLR; 3) Upregulate FXR, FGFR4, CYP7A1, BSEP, MRP2, and LXR	1) Inhibit lipid synthesis; 2) Promote β-oxidation; 3) Promote LDL-C degradation; 4) Promote bile acids synthesis and excretion	[187, 196]
16	*Cassiae semen*	Extract	↓TC, TG, LDL,	1) Improve intestinal microbiota composition and barrier damage; 2) Upregulate LDLR mRNA	1) Regulate gut microbiota; 2) Promote LDL-C degradation	[203, 204]
		Anthraquinone glycoside	↓ LDL; ↑HDL	1) Increase the PPARα expression; 2) Inhibit the SREBP-1c expression	1) Inhibit triglyceride synthesis; 2) Promote fatty acid oxidation	[206]
		1,8-Dihydroxyanthraquinone	↓TC, TG, LDL; ↑HDL	1) Upregulate CYP7A1; 2) Downregulate HMGCR	1) Inhibit cholesterol synthesis; 2) Promote cholesterol conversion into bile acids	[207]
17	*Canavaliae semen*	Bacillus subtilis-fermented extract	↓TG	1) Downregulate aP2, adiponectin, C/EBPα, PPARγ and FAS; 2) Upregulate PPARα, ACOX, LCAD, pHSL and ATG	1) Inhibit lipid biosynthesis; 2) Promote triglyceride hydrolysis and β-oxidation	[212]
		Total terpenoids; total flavonoids	↓TC, TG, LDL; ↑HDL	/	/	[213]
18	*Lablab semen album*	Extract	↓TC, TG, LDL, FFA	1) Downregulate Fsp27/Cidec, VLDLR, CD36, DGAT1 and DGAT2; 2) Increase adiponectin levels; 3) Regulate GL/FFA cycle and bile acid metabolism	1) Inhibit TG synthesis and fatty acid up-take; 2) Increase FFA oxidation; 3) Improve endogenous metabolites	[220, 221]
19	*Persicae semen*	Amygdalin	↓TC, TG, LDL; ↑HDL	/	/	[225, 226]
		Peach kernel oil	↓TC, TG, LDL; ↑HDL	/	/	[228]
20	*Portulacae herba*	Extract/powder	↓TC, TG, LDL, VLDL; ↑HDL	Upregulate the protein expression of PPAR-α and PPAR-γ	Promote lipolysis and fatty acid oxidation	[236, 239]

## Peels

### *Citri reticulatae pericarpium* (Chenpi)

*Citri reticulatae pericarpium* (Chenpi) is the dried mature peel of *Citrus reticulata* Blanco and its cultivars [31, 32]. Chenpi can be made into snack foods, beverages, tea, or used as cooking materials, seasonings, and spices [141]. It has pharmacological activities of promoting digestion, protecting the liver, anti-asthma, anti-cough, anti-inflammation, and anti-oxidation [142]. Approximately 140 chemical constituents have been separated and identified from Chenpi, and the main bioactive components are flavonoids, limonoids, alkaloids, and volatile oils [141, 143].

Flavonoids in Chenpi are mainly divided into flavonoid glycosides (hesperidin, naringin, etc.) and polymethoxyflavonoids (nobiletin, tangeretin, etc.) [144]. Polymethoxyflavonoids is the unique chemical composition of citrus. It is a low polarity fat-soluble substance, easily soluble in organic solvents such as hot ethanol and ethyl acetate, but hardly dissolves in water [145]. Studies have reported that Chenpi extracts, especially extracted with 95% ethanol and ethyl acetate, can significantly reduce the TC and LDL-C levels in serum of hyperlipidemia rats induced by fat emulsion. Pharmacodynamics-component correlation analysis showed that polymethoxyflavonoids might be the effective component in lowering blood lipids [146]. HMGCR is the rate-limiting enzyme of cholesterol biosynthesis [147]. The 95% ethanol extract of Chenpi can decrease the contents of TC and LDL-C by inhibiting the activity of HMGCR and regulating ApoB and ApoA1 [148]. It can also reduce the serum levels of TG and free fatty acids (FFA) via increasing the activity of triglyceride metabolic related enzymes ATGL, LPL and HL, and up-regulating the mRNA expressions of PPARγ and FXR [149]. Moreover, water extract of Chenpi can modulate the abundance and diversity of gut microbiota to improve serum lipid parameters and decrease body weight [150].

### *Zanthoxyli pericarpium* (Huajiao)

*Zanthoxyli pericarpium* is the dried pericarp of *Zanthoxylum schinifolium* Sieb. et Zucc. or *Z. bungeanum* Maxim. of family Rutaceae [31, 32]. There are many varieties of *Zanthoxyli Pericarpium* for both medicinal and edible purposes. In various parts of Asia, Africa and America, *Z. bungeanum* specie is used by locals in food preparation and as a raw medicinal material [151]. In China, Szechuan pepper is a popular variety commonly used in daily cooking due to its exceptional aroma and flavor [152]. The narcotic or anti-irritant properties render them effective for pain relief, especially in the treatment of toothache [153]. *Zanthoxyli pericarpium* contains chemical components including volatile oil, alkaloids, amides, coumarin, lignin, fatty acids, triterpene and sterols [154], which endows it with biological activities of antioxidation, anti-inflammation, antitumor, antibacterial, gastrointestinal system regulation, and hypolipidemia [155, 156].

Hydroxy-*α*-sanshool isolated from *Z. bungeanum* has been found to exert lipid-lowering and anti-obesity effects in hyperlipidemia rats. After supplementation for 4 weeks, the contents of TC, TG and LDL-C in serum and liver significantly decreased, while HDL-C increased. Furthermore, abdominal adipose tissues, liver adipocytes and levels of oxidative stress markers reduced. The underlying mechanism is to promote lipid metabolism and lipoprotein transformation by up-regulating the expression of PPARγ and ApoE [157].

## Rhizomes

### *Dioscoreae rhizoma* (Shanyao)

*Dioscoreae rhizoma*, also known as Chinese yam, is the dried rhizome of *Dioscorea opposita* Thunb. of Dioscoreaceae [31, 32]. The roots, tubers, and rhizomes of yam have been used as food and traditional medicine by indigenous people since pre-historic times [158]. In West Africa and Asia, yam tuber is usually boiled, fried, baked, roasted, or eaten raw [158]. Yam provides abundant nutritional benefits and contains plentiful chemical compounds including diosgenin, flavonoids, polysaccharides, phenols, saponins, tannins and alkaloids [159]. Studies suggest that yam possesses potential pharmacological activities such as lipid-lowering, immunomodulation, antioxidation, estrogen stimulation, angiotensin I-converting enzyme inhibition and trypsin inhibition [160].

Diosgenin, a steroid sapogenin isolated from yam, is considered as a natural precursor of steroidal drugs [161]. In Wistar rats fed with high-cholesterol diets, administration of 0.5% diosgenin for 6 weeks significantly increased serum HDL concentrations and fecal cholesterol contents, but decreased the levels of hepatic TC, TG and fecal bile acids. The potential lipid-lowering mechanism might be through activating the catabolic pathway of AMPK, thus inhibiting cholesterol absorption and facilitating cholesterol excretion [162]. Resistant starch is a component of starch that is not digested in the small intestine but fermented by the microbiota in colon and produces short-chain fatty acids [163]. In hyperlipidemic golden hamsters induced by a high-fat diet, supplementation of resistant starch (0.5 and 1.5 g/100 g) obtained from yam for 4 weeks significantly improved blood lipid profiles, including TC, TG, LDL-C and HDL-C, which was achieved by increasing the alpha diversity of gut microbiota (Table 1 and 2) [164].

**Table 2:** Animal models and interventions of MEPs in the treatment of dyslipidemia

No	MEPs	Intervention	Animal model	Dosage	Period	Control	Dosage	References
1	*Cinnamomi cortex*	Powder	Albino Rats	2 and 4 g/kg	30 days	/	/	[38]
		Extract	C57BL/6 J mice	1%	14 weeks	/	/	[39]
		Polyphenol	Wistar rats	100 mg/kg	12 weeks	/	/	[40]
		Extract	White mice	2 mg, 4 mg and 8 mg/20 g	28 days	/	/	[41]
		Extract	Albino Rats	250 and 500 mg/kg	7 days	Atorvastatin	10 mg/kg	[42]
2	*Chrysanthemi flos*	Extract	SD rats	0.2% and 0.4%	13 weeks	/	/	[50]
		Extract	SD rats	1, 2 and 4 g/kg	8 weeks	Fenofibrate	0.02 g/kg	[51]
		Flavonoids, luteolin and luteoloside	SD rats	100 mg/kg; 50 mg/kg; 25 mg / kg	6 weeks	Simvastatin	10 mg/kg	[53]
3	*Citri sarcodactylis fructus*	Polyphenols	mice	50 mg/kg	11 weeks	/	/	[63]
		Polyphenols	Wistar rats	20 mg/Kg	90 days	/	/	[64]
		Polyphenols	SD rats	10 mg/Kg	4 weeks	/	/	[65]
		Naringin	C57BL/6 J Mice	25, 50 and 100 mg/kg	8 weeks	/	/	[66]
4	*Crataegi fructus*	Extract	SD rats	50 and 100 mg/kg	4 weeks	/	/	[71]
		Extract	SD rats	5% and 10%	4 weeks	/	/	[72]
		Freeze-dried powder	ApoE-/- mice	1, 2, and 5 g/kg	12 weeks	/	/	[75]
		Concentrated juice	KunMing mice	10, 15, and 20 ml/kg	5 weeks	/	/	[76]
		Pectin penta-oligogalacturonide	KunMing mice	300 mg/kg	4 weeks	/	/	[77]
		Crude glycoprotein	KunMing mice	1.0, 1.5 and 2.0 g/kg	4 weeks	/	/	[78]
		Vitexin	C57BL/6 J mice	5 mg/kg	12 weeks	/	/	[79]
		Preparation	ApoE − / − mice	/	16 weeks	/	/	[80]
5	*Gardeniae fructus*	Extract	SD rats	25, 50, and 100 mg/kg	6 weeks	Metformin	100 mg/kg	[87]
		Geniposide	Nrf2 − / − C57BL/6 mice	50, 75 and 100 mg/kg	19 h	Fenofibrate	100 mg/kg	[88]
		Geniposide	C57BL/6 and ApoE − / − mice	50 mg/kg	13 weeks	/	/	[89]
		Geniposide	ApoE − / − mice	50 mg/kg	12 weeks	/	/	[90]
		Geniposide	ApoE–/– mice	50 and 100 mg/kg	4 weeks	/	/	[91]
		Genipin	SD rats	12.5 and 25 mg/kg	12 days	/	/	[93]
		Genipin	C57BL/6 J mice	5 and 20 mg/kg	9 weeks	Rosiglitazone	2 mg/kg	[94]
6	*Hippophae fructus*	Flavonoids	KunMing mice	100, 200 and 400 mg/kg	42 days	/	/	[100]
		Flavonoids	C57BL/6 mice	100 and 300 mg/kg	9 weeks	/	/	[101]
		Sterol	SD rats	100, 200 and 400 mg/kg	42 days	Simvastatin	3.5 mg/kg	[104]
7	*Mori fructus*	Extract	SD rats	4 g/kg	4 weeks	/	/	[110]
		Dried fruit	C57BL/6 J mice	100 and 300 mg/kg	3 months	/	/	[112]
		Polysaccharides	db/db mice	200, 500 and 800 mg/kg	8 weeks	/	/	[113]
		Polysaccharides	Wistar rats	400 mg/kg	7 weeks	/	/	[114]
8	*Mori folium*	Powder	SD rats	0.9, 0.6, and 0.3 g/kg	5 weeks	Atorvastatin	6.0 mg/kg	[121]
		Extract	SD rats	0.5% and 1%	4 weeks	/	/	[122, 123]
		Extract	Wistar rats	0.5%, 1% and 2%	10 weeks	/	/	[116]
		Flavonoids	db/db mice	180 mg/kg	7 weeks	Metformin	200 mg/kg	[124]
		Flavonoids	SD rats	50, 100 and 200 mg/kg	/	Fenofibrate	50 mg/kg	[125]
		Polyphenols, fiber	SD rats	0.8, 0.12, 0.48 and 0.6 g/kg	6 weeks	Orlistat	0.0324 g/kg	[126]
		Extract	C57BL/6 mice	0.5%, 1.0% and 2.0%	8 weeks	/	/	[127]
		Polysaccharides	C57BL/6 mice	200, 400 and 800 mg/kg	8 weeks	Orlistat	25 mg/kg	[128]
9	*Nelumbinis folium*	Powder	SD rats	600 mg/kg	18 weeks	/	/	[131]
		Nuciferine	SD rats	20 mg/kg	8 weeks	/	/	[133]
		Nuciferine	C57BL/6 mice	7.5, 15 and 30 mg/kg	8 weeks	Metformin	90 mg/kg	[134]
		Nuciferine	C57BL/6 J mice	0.30%	8 weeks	/	/	[136]
		Nuciferine	SD rats	10 mg/kg	8 weeks	Simvastatin	10 mg/kg	[137]
10	*Citri reticulatae pericarpium*	Extract	SD rats	5 g/kg	4 weeks	Simvastatin	4 mg/kg	[146]
		Extract	SD rats	1.25, 2.5 and 5 g/kg	4 weeks	Simvastatin	4 mg/kg	[148]
		Extract	SD rats	1.25, 2.5 and 5 g/kg	6 weeks	Ezetimibe	1 mg/kg	[149]
		Extract	C57BL/6 mice	5 and 10 g/kg	12 weeks	Simvastatin	2 mg/kg	[150]
11	*Zanthoxyli pericarpium*	Hydroxy-*α*-sanshool	Wistar rats	9, 18 and 36 mg/kg	4 weeks	Fenofibrate	18 mg/kg	[157]
12	*Dioscoreae rhizoma*	Diosgenin	Wistar rats	0.50%	6 weeks	/	/	[162]
		Resistant starch	golden hamsters	0.5, and 1.5 g/100 g	4 weeks	/	/	[164]
13	*Polygonati rhizoma*	Extract	SD rats	1, 2 and 4 g/kg	14 weeks	Resveratrol	40 mg/kg	[165]
		Extract	SD rats	4 g/kg	14 weeks	Simvastatin	1.8 mg/kg	[170]
		Polysaccharides	KunMing mice	200, 400, and 800 mg/kg	14 days	Simvastatin	30 mg/kg	[171]
		Polysaccharides	SD rats	120, 240, 480 mg/kg	14 weeks	Simvastatin	1.8 mg/kg	[172]
		Saponin	ICR mice	1, 1.5, and 2 g/kg	4 weeks	Metformin	0.5 g/kg	[173]
		Syringaresinol-di-o-β-d-glucoside	SPF mice	25, 50 and 75 mg/kg	2 weeks	/	/	[174]
14	*Astragali radix*	Total flavones	C57BL/6 J mice	5, 25 and 50 mg/kg	8 weeks	Metformin	0.15 g/kg	[182]
		Total flavones	ApoE − / − mice	10 and 20 mg/kg	16 weeks	/	/	[183]
		Astragaloside IV	SD rats	80 mg/kg	8 weeks	Metformin	200 mg/kg	[186]
15	*Puerariae radix*	Extract	C5BL/6 mice	100 and 300 mg/kg	16 weeks	Metformin	250 mg/kg	[193]
		Puerarin	SD rats	2 g/kg	16 weeks	/	/	[194]
		Puerarin	SD rats	100 mg/kg	8 weeks	/	/	[195]
		Polysaccharides	db/db mice	100 and 200 mg/kg	6 weeks	Rosiglitazone	10 mg/kg	[187]
		PL-S2	Wistar rats	50 mg/kg	3 weeks	Simvastatin	8 mg/kg	[196]
16	*Cassiae semen*	Extract	SD rats	10 g/kg	4 weeks	Atorvastatin	10 mg/kg	[201]
		Extract	SD rats	54, 162 and 486 mg/kg	4 weeks	Atorvastatin	10 mg/kg	[202]
		Total aglycones (TA), rubrofusarin-6-β-gentiobioside (RG) and aurantio-obtusin (AO)	C57BL/6 mice	TA 10 g/kg, RG 20 mg/kg, and AO 20 mg/kg	3 weeks	/	/	[203]
		Extract	Wistar rats	0.5, 1, and 2 g/kg	6 weeks	Metformin	0.2 g/kg	[204]
		Anthraquinone glycoside	SD rats	5, 10 and 20 mg/kg	6 weeks	Polyene phosphatidylcholine	23 mg/kg	[206]
		1,8-Dihydroxyanthraquinone	mice	5 mg/kg	6 weeks	/	/	[207]
17	*Canavaliae semen*	Total terpenoids and total flavonoids	Wistar rats	400 mg/kg	3 weeks	Glibenclamide	5 mg/kg	[213]
		Protein extract	SD rats	4 and 6 g/200 g	2 weeks	/	/	[215]
18	*Lablab semen album*	Extract	C57BL/6 J mice	25 mg/kg	9 weeks	Milk thistle	100 mg/kg	[220]
		Extract	C57BL/6 J mice	25, 50 and 100 mg/kg	9 weeks	Milk thistle	100 mg/kg	[221]
19	*Persicae semen*	Amygdalin	LDLR-/- mice	1, 3 and 10 mg/kg	4 weeks	/	/	[225]
		Amygdalin	ApoE-/- mice	0.04 and 0.08 mg/kg	12 weeks	Simvastatin	2.57 mg/kg	[226]
		Peach kernel oil	ApoE-/- mice	2 and 5 g/kg	8 weeks	Simvastatin	5 mg/kg	[228]
20	*Portulacae Herba*	Extract	Wistar rats	400 mg/kg	4 weeks	Atorvastatin	10 mg/kg	[235]
		Extract	Wistar rats	10 g/kg	4 weeks	/	/	[236]
		Extract	albino rats	5% and 10%	8 weeks	/	/	[237]
		Extract	Wistar rats	0.50%	4 weeks	/	/	[238]
		Extract	C57BL/6 mice	5% and 10%	12 weeks	/	/	[239]

### *Polygonati rhizoma* (Huangjing)

*Polygonati rhizoma* is the dried rhizome of *Polygonatum kingianum* coll. et Hemsl, *P. sibiricum* Red. or *P. cyrtonema* Hua of family Liliaceae [32]. In Asia, Europe and North America, it has traditionally been used as herbal medicine and nutrient food to treat diabetes, cough, fatigue and feebleness [165, 166]. *Polygonati rhizoma* contains many chemical ingredients, mainly including polysaccharides, steroidal saponins, flavonoids, alkaloids, lignin and amino acids [167]. Attributed to these compounds, *Polygonati rhizoma* has pharmacological effects of anti-aging, anti-tumor, immunomodulation, antibacterial, hypoglycemic and hypolipidemia [168, 169].

High-fat diet leads to NAFLD, administration of *Polygonati rhizoma* extract (4 g/kg) for 14 weeks restored disordered blood lipid levels of rats, including TC, LDL-C and HDL-C. It can upregulate and downregulate the mRNA expression of CPT1 and uncoupling protein 2 (UCP2), respectively, as well as modulate endogenous metabolites [165, 170]. Meanwhile, NAFLD was ameliorated due to the enhancement of mitochondrial antioxidant function and fatty acid β-oxidation [165].

Polysaccharides and saponins are likely to be the effective compounds for dyslipidemia treatment. In hyperlipidemia mouse model induced by intraperitoneal injection of 75% fresh egg yolk emulsion, *Polygonati rhizoma* polysaccharides can reduce the contents of TC, TG, and LDL-C in serum whereas increase HDL-C. Its lipid-lowering mechanism is related to modulating the mRNA and protein expressions of PPARs and SREBP-1c [171]. Besides dyslipidemia, other metabolic diseases such as diabetes and obesity can also be ameliorated by *Polygonati rhizoma*. The polysaccharides (120, 240 and 480 mg/kg) treatment for 14 weeks can reduce the levels of blood lipids, glucose and weight gain by acting on the intestinal flora. It can regulate the composition, abundance and diversity of gut microbiota, decrease intestinal epithelial cell permeability and inhibit lipase entry into the entero-hepatic circulation [172].

Saponin, the essential active component of *Polygonati rhizoma*, was found to decrease serum lipid profiles and glucose in type 2 diabetes mellitus (T2DM) mice induced by a high-fat diet and streptozotocin solution injection. Further analysis suggested that the improvement of the gut microbiota might be responsible for the restoration of metabolic disorders [173]. Syringaresinol-di-O-β-D-glucoside, a phenolic compound isolated from *Polygonati rhizoma*, is able to lower the levels of TC, TG, LDL-C, VLDL-C and FFA in serum of diabetic mice, decrease the levels of oxidative stress indexes and increase insulin sensitivity [174].

## Roots

### *Astmgali radix* (Huangqi)

*Astragali radix* is the dried root of *Astragalus membranaceus* (Fisch.) Bge. var. *mongholicus* (Bge.) Hsiao or *Astragalus membranaceus* (Fisch.) Bge. of family Leguminosae [31, 32]. Northern China and Mongolia are the origins of *Astragali radix*, which is also cultivated in other temperate regions of the world such as Siberia and North Korea [175]. Due to its pharmacological activity, it is commonly used as a crude drug in Oriental medicine [176]. For example, *Astragali radix* is a popular tonic herb for nourishing qi and blood, as well as promoting urination to relieve edema in TCM [177]. In addition to medicinal uses, it can also be made into herbal tea, beverages, and cooking dishes for daily consumption [178]. There are more than 100 compounds identified from *Astragali radix*, including saponins (astragaloside, acetytastragaloside, isoastragaloside, etc.), flavonoids (Calycosin 7-O-glucoside, kaempferol, quercetin, isorhamnetin, etc.), polysaccharides, amino acids and trace elements [179, 180]. Studies indicate that *Astragali radix* has pharmacological effects of anti-oxidation, hypolipidemia, hypotension, anti-inflammation, immune regulation, cardiovascular protection and anti-hepatic fibrosis [175, 179, 181].

It was found that flavones derived from *Astragali radix* could reduce the levels of cholesterol and triglyceride, while increasing the content of HDL-C both in vivo and in vitro. This beneficial effect is achieved by regulating the expression of FXR, G protein-coupled bile acid receptor (TGR5), CYP7A1 and ASBT proteins involved in bile acid metabolism [182]. Furthermore, the flavones can downregulate the expression of lipid genesis genes FAS and SREBP-1c, while upregulating the levels of fatty acid oxidation genes AMPKα and CPT1A. As a result, the TC, TG, LDL and VLDL contents in serum of ApoE-/- mice decreased, HDL-C level increased, and the progress of atherosclerosis attenuated [183].

Astragaloside IV, a small molecular bioactive saponin isolated from *Astragali radix* [184], was able to downregulate the expression of adipogenesis genes SREBP-1, ACC1, FAS and SCD1 via activating AMPK and ACC phosphorylation. Meanwhile, lipid accumulation, endoplasmic reticulum stress and hepatic steatosis induced by FFA in hepatocytes were alleviated [185]. In T2DM rat model, Astragaloside IV protect against diabetic cardiomyopathy by improving the lipid accumulation in cardiomyocytes, decreasing the contents of TC, TG in serum and FFA in tissue, and elevating the plasma HDL-C level [186].

### *Puerariae radix* (Gegen)

*Puerariae radix*, the dried root of the leguminous plant *Pueraria lobata* (Willd.) Ohwi or *Pueraria thomsonii* Benth, has been traditionally used as a source of medicine and food in China, Japan and Korea [187]. There are two different kinds of Chinese *Puerariae radix*, one is called Yege (*Puerariae lobatae radix*) and the other is Fenge (*Puerariae thomsonii radix*). Both of them contain isoflavones, the major bioactive constituents, including puerarin, daidzin, daidzein, genistin, genistein and other compounds [188]. Although used interchangeably in clinical practice, there are still distinctions between Yege and Fenge [189]. It is considered that Yege has better medicinal value attributes to its higher isoflavones, while Fenge is more suitable for eating due to being abundant in starch and sweet in taste [31, 190]. Currently, *Puerariae radix* is widely used to treat diseases such as hyperlipidemia, hypertension, coronary heart disease, liver injury, fever and diarrhea [191].

*Puerariae radix* plays a role in treating hyperlipidemia and other metabolic diseases through multiple potential mechanisms [192]. In obese mice model induced by a high-fat diet, *Puerariae radix* extract (100 or 300 mg/kg) administration for 16 weeks improved the levels of TC and HDL-C, glucose tolerance and liver lipid accumulation. The increased expression of peroxisome proliferator-activated receptor-γ coactivator (PGC)-1α proteins mediated by AMPK activation might be responsible for these effects [193].

Puerarin, the main component of *Puerariae radix*, improved dyslipidemia by decreasing the mRNA expression of lipogenic genes including SREBP-1c, FAS, SCD1 and HMGCR, while increasing the phosphorylation of AMPK and ACC, which lead to the reduction of TC content and lipid accumulation in HepG2 cells (Table 1 and 3) [194]. In rats model of type 2 diabetic induced by a high-fat diet combined with low-dose streptozotocin, puerarin treatment decreased serum TC, TG, LDL-C and glucose levels. It can downregulate the mRNA expression of SREBP-1c and SCD1, upregulate CPT1 and acyl-coenzyme A oxidase (ACOX), and restore the activity of hepatic lipase. As a result of glycolipid metabolism and oxidative stress improvement, hepatic steatosis was also ameliorated [195].

**Table 3:** Cell models and interventions of MEPs in the treatment of dyslipidemia

No	MEPs	Intervention	Cell model	Concentration	Duration	References
1	*Chrysanthemi flos*	Extract	HUVECs	50, 100 and 200 μg/mL	24 h	[51]
2	*Citri sarcodactylis fructus*	Extract	3T3-L1 cells	0.85 and 0.56 mg/ml	5 days	[60]
		Extract and main components	HepG2 and Caco-2 Cells	50 and 100 µg/mL	24 h	[61]
3	*Crataegi fructus*	Extract	3T3-L1 cells	50, 100, and 200 ug/mL	24 h	[72]
		Vitexin	3T3-L1 cells	10 and 50 µM	8 days	[79]
4	*Gardeniae fructus*	Geniposide	HepG2	0, 65, 130, 260, 390 and 520 μmol/L	24 h	[88]
		Geniposide	HepG2 cells and Caco2 cells	100 μM	12 and 24 h	[89]
		Geniposide	RAW264.7 macrophage cells	2.5, 5, 10, 20, 40 and 80 μM	24 h	[90]
		Geniposide	RAW264.7 macrophage cells	50, 100, and 200 μg/ml	24 h	[91]
		Genipin	Primary hepatocytes	20 μM	24 h	[94]
5	*Hippophae fructus*	Flavonoids	HL7702 cells	5, 10, 20, 40 and 80 μg/mL	24 h	[102]
		Kaempferol and kaempferide	HepG2 Cells	5, 10 and 20 μM	48 h	[103]
6	*Mori fructus*	Extract	3T3-L1 cells	10, 50, 100, and 500 ng/mL	7 days	[111]
7	*Mori folium*	Flavonoids	L6 skeletal muscle cells	5, 10, 20, 40 and 80 µg/ml	24 h	[124]
		Flavonoids	HepG2 cells	1, 5, 10, 30, 60, 90, 150, and 180 μmol /L	24 h	[125]
		Extract	HepG2 cells	2 mg/mL	24 h	[127]
		Polysaccharides	HepG2 cells	25, 50, 100, 150 and 200 μg/mL	24 h	[128]
8	*Nelumbinis folium*	Nuciferine	Caco-2 and HT-29 cells	0, 25, 50, 100 and 200 μM	24 h	[136]
		Nuciferine	3T3-L1 preadipocytes	0, 2.5, 5, 10 and 20 μM	24, 48, 72, 96 and 120 h	[138]
		Nuciferine	THP-1 cells	5, 10 and 20 μmol/L	24 h	[139]
		Nuciferine	THP-1 cells	2.5, 5, 10, and 20 μmol/L	24 h	[140]
9	*Dioscoreae rhizoma*	Diosgenin	C2C12 cells	0, 20, 40 and 80 μM	3 h	[162]
10	*Astragali radix*	Total flavones	HepG2 cells	0, 2.5, 5, 10, 20 and 40 μg/ml	24 h	[182]
		Total flavones	HUVECs, RAW264.7, THP-1 cells and peritoneal macrophages	6, 12 and 24 μg/ml	12 h	[183]
		Astragaloside iv	HepG2 cells	50, 100, and 200 μg/mL	24 h	[185]
11	*Puerariae radix*	Extract	C2C12 cells	0.2 and 0.5 mg/mL	24 h	[193]
		Puerarin	HepG2 cells	75 and 150 μM	24 h	[194]
12	*Canavaliae semen*	Extract	3T3-L1 cells	100, 200, 400, and 1000 μg/mL	48 h	[212]
13	*Persicae semen*	Amygdalin	bone marrow-derived macrophages	25, 50, 100, 200, 400, and 800 μg/ml	24 h	[226]
		Peach kernel oil	HUVECs and RAW264.7 macrophage cells	0.01, 0.05, 0.1, 0.15, and 0.2 μg/mL; 50, 100 and 200 µg/mL	24 h	[228]

Polysaccharides isolated from *Puerariae radix* administered for 6 weeks were found to increase the content of HDL-C in serum, but decrease the levels of TG, TC, LDL-C, and FFA. The underlying mechanism is through upregulating the mRNA expression of PPARα and LDLR while downregulating SREBP-1 and ACC [187]. Bile acids play a pivotal role in the lipid metabolism. The novel homogeneous polysaccharide PL-S2 derived from *Puerariae radix* exerts hypolipidemic function by facilitating bile acids synthesis and excretion mediated via the FXR signaling pathway [196].

## Seeds

### *Cassiae semen* (Juemingzi)

**Fig. 3:**
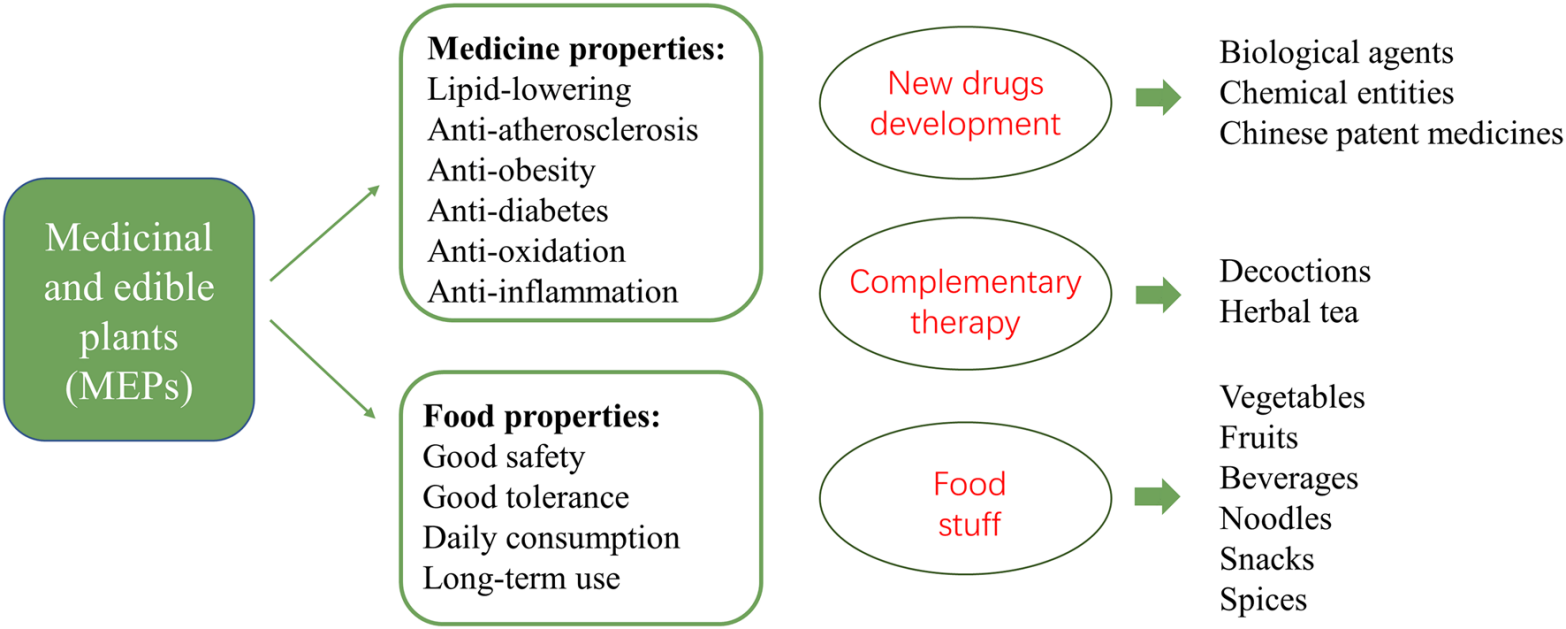
MEPs have the dual properties and advantages of medicine and food. In addition to regulating metabolism, MEPs also have other beneficial effects and can protect from cardiovascular diseases, which have great potential for the development of new drugs, complementary therapies and functional foods

*Cassiae semen*, also known as cassia seed, is the dried mature seed of *Cassia obtusifolia* L. or *C. tora* L. (Cassia minor) of Leguminosae [31, 32]. It grows in tropical Asian countries with strong vitality and is widely cultivated in Korea and China [197]. Cassia seed is popular as a functional roasted tea in China. TCM believes that it can nourish the liver, improve eyesight, relieve constipation and alleviate headache. It contains anthraquinones, naphthopyranones, fatty acids, polysaccharides, and other chemical ingredients [198]. Except for pharmacological activities of antihypertension, lowering blood sugar, relieving bowels, and antioxidation, it has shown potential therapeutic effects on dyslipidemia [199, 200], which is one of the promising MEPs for the development of lipid-lowering drugs and its derivates (Fig. 3).

Studies reported that cassia seed extract could effectively improve lipid profiles of hyperlipidemia rats, and reduce the contents of TC, TG and LDL-C in serum [201, 202]. The mechanism might be through regulating the gut microbiota [203]. In addition, the ethanol extract of cassia seed decreased the contents of TC and TG in blood and upregulated the mRNA expression of LDLR in a dose-dependent manner [204]. Cassia seed contains a variety of chemical components, among which anthraquinones are the most important pharmacologically active ingredients for lipid-lowering [198, 205]. Research indicated that the anthraquinone glycoside isolated from cassia seed could regulate lipid metabolism by increasing PPARα expression and inhibiting SREBP-1c expression in the liver tissue of rats [206]. Moreover, the 1,8-dihydroxyanthraquinone separated from cassia seed can upregulate and downregulate the protein expression of CYP7A1 and HMGCR, respectively, thereby modulating cholesterol metabolism and reducing the contents of TG, TC, and LDL-C in serum of hyperlipidemia mice [207].

### *Canavaliae semen* (Daodou)

*Canavaliae semen*, also called sword bean or Jack bean, is the mature dried seeds of the *Canavalia gladiata* (Jacq.) DC of family Legume [31, 32]. In Asia, young pods and seeds of sword bean are consumed as green vegetables with desirable nutrients of protein, fatty acids, amino acids, minerals and starch. In Latin America, roasted seeds are usually used to prepare a coffee-like beverage [208]. Sword bean contains phenols, flavonoids, urease, concanavalin, gallic acid, and erythrocyte lectin. These bioactive compounds endow it with antioxidant, antibacterial, antiangiogenic, immunomodulatory and anticancer activities [209, 210]. In addition, it has potential treatment effects on metabolic diseases such as dyslipidemia, obesity and diabetes.

In states of overnutrition, excess calories are stored in the form of triglycerides and accumulated in white adipose tissue, leading to dyslipidemia and obesity [211]. The bacillus subtilis-fermented white sword bean extract can phosphorylate AMPK in the early stage of adipocyte differentiation, inhibit the mRNA expression of aP2 and adiponectin, as well as reduce the protein levels of C/EBPα, PPARγ, and FAS, which results in the decrease of TG accumulation. Concurrently, it can increase the mRNA expression of PPARα, ACOX, and long-chain acyl coenzyme A dehydrogenase (LCAD) as well as the protein levels of pHSL and ATGL, to promote lipolysis in mature 3T3-L1 adipocytes [212]. Total triterpenoids and total flavonoids in sword bean have been revealed to improve serum lipid profiles, body weight, blood glucose and antioxidant indexes [213, 214]. Moreover, in hypercholesterolemic rats, sword bean protein extract (2 or 3 g/100 g) intervention for 2 weeks was found to lower the levels of TC, TG, LDL-C and VLDL-C in blood whereas increasing the content of HDL-C [215].

### *Lablab semen album* (Baibiandou)

*Lablab semen album*, commonly known as white hyacinth bean, is the dried mature seed of *Dolichos lablab* L. belongs to family Fabaceae [31, 32]. For centuries, it has been traditionally used in Asian medicine such as China and South Korea to treat gastrointestinal disorders. In India, cooked hyacinth bean pods are eaten to alleviate diarrhea, nausea, vomiting and poor appetite [216, 217]. White hyacinth bean contains chemical components including flavonoids, saponins, coumarins, terpenes, alkaloids, tannins, alcohols, phenols, steroids and essential oils [218]. Pharmacological studies have shown that it has hypolipidemic, hypoglycemic, anti-inflammatory, antioxidant, and hepatoprotective properties [219].

In obese mice with dyslipidemia, the dietary administration of hyacinth bean (25 mg/kg/day) for 9 weeks significantly decreased the levels of TC, TG, LDL-C and FFA in serum as well as alleviated hepatic steatosis compared to model group. Metabolomics results indicated that the attenuation of amino acid, lipid, glucose, bile acid metabolism and glycerolipid/free fatty acid (GL/FFA) cycle is the potential mechanism for improving dyslipidemia and obesity [220]. Furthermore, white hyacinth bean could ameliorate lipid profiles and NAFLD via down-regulating the expression of mRNA and protein that mediated fatty acid uptake and lipid droplet accumulation [221]. Nonetheless, which chemical components play a role in lipid-lowering still needs further study.

### *Persicae semen* (Taoren)

*Persicae semen*, also called peach kernel, is the dried and mature seed of *Prunus persica* (L.) Batsch of Rosaceae or *P. davidiana* (Carr.) Franch. of Yamada [31, 32]. Peach kernel is conventionally used to activate blood circulation, remove stasis, loosen the bowel, and relieve constipation [222]. Modern pharmacological studies have found that peach kernel contains a variety of chemical components, including volatile oils, cyanogenic glycosides, flavonoids, sterols, aromatic glycosides, fatty acids, phenylpropanoids, nucleosides and trace elements [223]. Biological activities such as cardio-cerebral vascular system protection, anti-inflammation, anti-tumor, immunomodulation, liver and kidney protection have been found in peach kernel [224].

Amygdalin, the main cyanogenic glycoside compound of peach kernel, has the functions of improving dyslipidemia and atherosclerosis. In LDLR-/- mice fed with a high-fat and high-cholesterol diet, amygdalin supplementation decreased the levels of TC, TG and LDL-C in serum whereas increased HDL-C. In addition, the inflammatory reaction and the development of atherosclerosis were attenuated [225]. In high-fat diet ApoE-/- mice, the blood lipid profiles, body weight, inflammatory cytokines, and atherosclerotic plaque area were all decreased after injection of amygdalin at the concentration of 0.08 or 0.04 mg/kg for 12 weeks [226]. Similar effects have also been found in peach kernel oil, which contains unique fatty acids including oleic acid (ω-9) and linoleic acid (ω-6) that are beneficial to the human body [227]. The administration of peach kernel oil reduced TC, TG and LDL-C levels whereas elevated HDL-C levels in mice serum. Moreover, the formation of atherosclerotic plaque was inhibited by down-regulating the expression of inflammatory genes and proteins [228].

## Whole herbs

### *Portulacae herba* (Machixian)

*Portulacae Herba*, also known as purslane, is the herbaceous weed of *Portulaca oleracea* L. belonging to the family Portulacaceae [31, 32]. It is an annual herb widespread in many countries and areas such as China, India, France, and Spain, usually eaten as a potherb with succulent leaves [229]. As a traditional medicinal herb, purslane possesses pharmacological properties including anti-inflammation, antibacterial, antioxidation, hypolipidemia, hypoglycemia, and hepatoprotection [229, 230]. It can be used to treat dermatosis, gynecological diseases and intestinal bacterial infections [231]. There are abundant bioactive ingredients in purslane such as flavonoids, polysaccharides, phenolic acids, alkaloids, triterpenoids, and essential fatty acids [232]. Besides, purslane riches in essential ω-3 and ω-6 fatty acids, ascorbic acid, α-tocopherol and β-carotene [233], which have beneficial effects on cardiovascular disease, diabetes, cancer, dementia, depression, visual and neurological development [234].

Studies reported that purslane extract has an excellent hepatoprotective property and lipid-lowering effects. It can reduce the levels of TG (26.99%), TC (10.91%), LDL (16.41%) and liver damage makers in dyslipidemia rats induced by a high-fat cafeteria food [235]. Furthermore, purslane is a promising natural product to prevent glycolipid metabolism disorder. In hypercholesterolemia combined with diabetes rats induced by a cholesterol-enriched diet and streptozotocin injection, the plasma parameters of TC, TG, LDL-C, VLDL-C and glucose decreased whereas HDL-C increased after purslane aqueous extract (1 g/100 g) administration for 28 days [236]. Similar lipid and glucose modulating effects were also discovered in purslane-supplemented rat models of diabetes and hypercholesterolemia, respectively, accompanied by improvements in insulin resistance and liver function [237, 238]. Flavonoids, phenolic compounds and omega-3 fatty acids are likely to be the effective ingredients [237]. The underlying mechanism might be through up-regulating protein expression levels of PPARα, glucose transporter (GLUT) 4 and PPARγ [239].

## Conclusion and perspective

In general, MEPs, including the extract and bioactive compounds, can regulate the concentrations of serum TG, TC, LDL-C and HDL-C to modify dyslipidemia. As shown in Table 1, the main effective components of MEPs for dyslipidemia treatment include flavonoids (kaempferol, naringin, quercetin, luteolin), isoflavones (puerarin), saponins (astragaloside IV, diosgenin), iridoid glycosides (geniposide, genipin), alkaloids (nuciferine), polysaccharides (pectin), sterols, polyphenols, anthraquinones and other bioactive components. The lipid regulation mechanism involves the whole process of lipid absorption, synthesis, transport, decomposition and excretion: (1) MEPs decrease intestinal epithelial cell permeability and inhibit lipid absorption. (2) MEPs inhibit de novo cholesterol biosynthesis, fatty acid uptake and triglyceride synthesis. (3) MEPs promote lipid catabolism by increasing cholesterol efflux and accelerating fatty acid oxidation. (4) MEPs regulate enterohepatic circulation of bile acids to decrease cholesterol. They can promote the conversion of cholesterol into bile acids and inhibit the bile acids reabsorption. (5) MEPs promote lipid transport and distribution by regulating apolipoprotein, HDL-C formation and reverse cholesterol transport. (6) MEPs modulate intestinal flora and relieve insulin resistance to improve lipid metabolism disorder (Fig. 1, Fig. 2 and Table 1). Notably, they can not only regulate lipid metabolism, but also possess potential benefits of lowering blood sugar, anti-obesity, resist atherosclerosis, antioxidation, and anti-inflammation, which contribute to the prevention of cardiovascular diseases. Based on this, the role of MEPs in the treatment of metabolic syndrome and cardiovascular disease deserves further exploration.

However, there are a large number of chemical ingredients in MEPs, and more attention should be paid to specific molecules rather than simple water or ethanol extracts in the future, which is conducive to the research and development of new drugs. For example, flavonoids and saponins include many molecules, but which one of them plays a role in the treatment of dyslipidemia needs further analysis and verification. Moreover, clinical trials, such as randomized controlled studies or cohort studies, are required to further demonstrate whether molecular compounds proven to be effective in vivo or in vitro respond similarly in humans, and at what doses and durations of treatment. After that, the effective components in MEPs can be isolated and optimized to develop Chinese patent medicines, biological agents or chemical entities. Moreover, in clinical practice, these MEPs can be properly formulated into decoctions or tea substitutes as a supplementary or alternative treatment, which can produce synergistic effects with conventional lipid-lowering drugs (Fig. 3).

Humans have always relied on plants for food and medicine since ancient times. MEPs, widely distributed around the world, are derived from natural plants and have been used in traditional medicine for thousands of years to treat diseases, strengthen physical fitness and improve quality of life through holistic regulation [112]. Given their food characteristics, they can be eaten directly, or made into food stuff. Intriguingly, they also have significant lipid-lowering effects with incomparable advantages, such as high efficiency, non-toxicity, easy access and long-term use, which provide a level of safety rarely achieved by allopathic drugs. In the daily diet, dyslipidemia population can moderately increase the intake of vegetables such as yam, white hyacinth bean or purslane, and fruits such as mulberry, bergamot or hawthorn. In addition, condiments or spices like Huajiao or Chenpi can be used when cooking, and *Polygonati rhizoma* or *Astragali radix* can be added when boiling soup. Besides, MEPs can also be processed into food products including cookies, candies, yogurts, and noodles for daily consumption. Drugs and diet have a strong impact on the occurrence and development of dyslipidemia. Whether for new drug development or complementary therapy, MEPs are optimal candidates and deserve further study.

## Data Availability

Not applicable.

## Abbreviations

ABCA1: ATP binding cassette subfamily A member 1
ABCG5: ATP binding cassette subfamily G member 5
ACAT: Acetyl-CoA acetyltransferase
ACC: Acetyl-CoA carboxylase
ACLY: ATP- citrate lyase
AGPAT: 1-Acylglycerol-3-phosphate o-acyltransferase
AMPK: Adenosine monophosphate-activated protein kinase
ApoA: Apolipoprotein A
ASBT: Apical sodium-dependent bile acid transporter
C/EBP: CCAAT/enhancer binding protein
CETP: Cholesteryl ester transfer protein
CPT1: Carnitine palmitoyltransferase 1
CYP2E1: Cytochrome P450 2E1
CYP7A1: Cholesterol 7 alpha-hydroxylase
FAS: Fatty acid synthase
FXR: Farnesoid X receptor
HDL-C: High-density lipoprotein cholesterol
HL: Liver lipase
HMGCR: Hydroxymethyl glutaryl-CoA reductase
LCAD: Long-chain acyl coenzyme A dehydrogenase
LCAT: Lecithin cholesterol acyltransferase
LDL-C: Low-density lipoprotein cholesterol
LDLR: Low-density lipoprotein receptor
LPL: Lipoprotein lipase
LXRα: Liver X receptor α
NPC1L1: Niemann-Pick C1 Like 1
pCEH: Pancreatic cholesterol ester hydrolase
PPARα: Peroxisome proliferator-activated receptors α
SCD1: Stearoyl-CoA desaturase 1
SREBP: Sterol regulatory element-binding protein
TC: Total cholesterol
TG: Triglyceride
TGR5: G protein-coupled bile acid receptor
UCP2: Uncoupling protein 2

## References

[1] Ference BA, Kastelein JJP, Catapano AL (2020). Lipids and lipoproteins in 2020. JAMA.

[2] Shengshou H (2021). China TWCotRoCHaDi. Report on cardiovascular health and diseases burden in China: an updated summary of 2020. Chin Circ J.

[3] Defesche JC, Gidding SS, Harada-Shiba M, Hegele RA, Santos RD, Wierzbicki AS (2017). Familial hypercholesterolaemia. Nat Rev Dis Primers.

[4] Murray CJ, Lopez AD (2013). Measuring the global burden of disease. N Engl J Med.

[5] Visseren FLJ, Mach F, Smulders YM, Carballo D, Koskinas KC, Back M (2021). 2021 ESC Guidelines on cardiovascular disease prevention in clinical practice. Eur Heart J.

[6] NCDRF Collaboration (2020). Repositioning of the global epicentre of non-optimal cholesterol. Nature.

[7] Mortensen MB, Nordestgaard BG, Afzal S, Falk E (2017). ACC/AHA guidelines superior to ESC/EAS guidelines for primary prevention with statins in non-diabetic Europeans: the Copenhagen general population study. Eur Heart J.

[8] Rossello X (2021). Lifetime risk estimation in atherosclerotic cardiovascular disease: where inflammation meets lipoprotein(a). J Am Coll Cardiol.

[9] Ibanez B, Fernandez-Ortiz A, Fernandez-Friera L, Garcia-Lunar I, Andres V, Fuster V (2021). Progression of early subclinical atherosclerosis (PESA) study: JACC focus seminar 7/8. J Am Coll Cardiol.

[10] Collins R, Reith C, Emberson J, Armitage J, Baigent C, Blackwell L (2016). Interpretation of the evidence for the efficacy and safety of statin therapy. Lancet.

[11] Pergolizzi JV, Coluzzi F, Colucci RD, Olsson H, LeQuang JA, Al-Saadi J (2020). Statins and muscle pain. Expert Rev Clin Pharmacol.

[12] Lv S, Yu H, Liu X, Gao X (2021). The study on the mechanism of hugan tablets in treating drug-induced liver injury induced by atorvastatin. Front Pharmacol.

[13] Mach F, Baigent C, Catapano AL, Koskinas KC, Casula M, Badimon L (2020). 2019 ESC/EAS Guidelines for the management of dyslipidaemias: lipid modification to reduce cardiovascular risk. Eur Heart J.

[14] Soppert J, Lehrke M, Marx N, Jankowski J, Noels H (2020). Lipoproteins and lipids in cardiovascular disease: from mechanistic insights to therapeutic targeting. Adv Drug Deliv Rev.

[15] Huang K, Zhang P, Zhang Z, Youn JY, Wang C, Zhang H (2021). Traditional Chinese medicine (TCM) in the treatment of COVID-19 and other viral infections: efficacies and mechanisms. Pharmacol Ther.

[16] Wang S, Fu JL, Hao HF, Jiao YN, Li PP, Han SY (2021). Metabolic reprogramming by traditional Chinese medicine and its role in effective cancer therapy. Pharmacol Res.

[17] Liu C, Huang Y (2016). Chinese herbal medicine on cardiovascular diseases and the mechanisms of action. Front Pharmacol.

[18] Xie G, Tang X, Liang X, Liu H, Zhang S (2020). The origination, connotation, and definition of one root of medicine and food. Mod Chin Med..

[19] Yang M, Sheng P (2021). Medicinal and edible resources in Xinjiang:current status and prospects. Chin J Exp Tradit Med Formulae.

[20] Pollastro F, Minassi A (2021). Exploring the universe of natural products: recent advances in synthesis, isolation and structural elucidation. Plants (Basel).

[21] Yuan H, Ma Q, Ye L, Piao G (2016). The traditional medicine and modern medicine from natural products. Molecules.

[22] Atanasov AG, Zotchev SB, Dirsch VM, Supuran CT (2021). Natural products in drug discovery: advances and opportunities. Nat Rev Drug Discov.

[23] Yang G, Su F, Chen M (2021). Origin and prospect of homology medicine and food. Mod Chin Med.

[24] Mollazadeh H, Mahdian D, Hosseinzadeh H (2019). Medicinal plants in treatment of hypertriglyceridemia: A review based on their mechanisms and effectiveness. Phytomedicine.

[25] El-Tantawy WH, Temraz A (2019). Natural products for controlling hyperlipidemia: review. Arch Physiol Biochem.

[26] Zhang Y, Kishi H, Kobayashi S (2018). Add-on therapy with traditional Chinese medicine: An efficacious approach for lipid metabolism disorders. Pharmacol Res.

[27] Hunter PM, Hegele RA (2017). Functional foods and dietary supplements for the management of dyslipidaemia. Nat Rev Endocrinol.

[28] Xie W, Zhao Y, Du L (2012). Emerging approaches of traditional Chinese medicine formulas for the treatment of hyperlipidemia. J Ethnopharmacol.

[29] National Health Commission of P. R. China. Management approach of food and Chinese medicine homologous catalogue according to tradition. 2014. http://www.nhc.gov.cn/wjw/yjzj/201411/67ac54fb05ed46929adc63f2db31d4bf.shtml. Accessed on 26 Apr 2022.

[30] National Health Commission of P. R. China. List of 9 pilot food and Chinese medicine homologous substances according to tradition. 2020. http://www.nhc.gov.cn/sps/s7885/202001/1ec2cca04146450d9b14acc2499d854f.shtml. Accessed on 26 Apr 2022.

[31] Huang L, Chen M (2021). Interpretation of medicine and food homologous substances.

[32] Pharmacopoeia Committee of P. R. China (2020). Pharmacopoeia of People’s Republic of China.

[33] Shalaby MA, Saifan HY (2014). Some pharmacological effects of cinnamon and ginger herbs in obese diabetic rats. J Intercult Ethnopharmacol.

[34] Ranasinghe P, Perera S, Gunatilake M, Abeywardene E, Gunapala N, Premakumara S (2012). Effects of Cinnamomum zeylanicum (*Ceylon cinnamon*) on blood glucose and lipids in a diabetic and healthy rat model. Pharmacognosy Res.

[35] Hamidpour R, Hamidpour M, Hamidpour S, Shahlari M (2015). Cinnamon from the selection of traditional applications to its novel effects on the inhibition of angiogenesis in cancer cells and prevention of Alzheimer's disease, and a series of functions such as antioxidant, anticholesterol, antidiabetes, antibacterial, antifungal, nematicidal, acaracidal, and repellent activities. J Tradit Complement Med.

[36] Liu Y, An T, Wan D, Yu B, Fan Y, Pei X (2020). Targets and mechanism used by cinnamaldehyde, the main active ingredient in Cinnamon, in the treatment of breast cancer. Front Pharmacol.

[37] Kuete Victor, Mbaveng AT (2017). Cinnamon Species. Medicinal spices and vegetables from Africa: therapeutic potential against metabolic, inflammatory, infectious and systemic diseases.

[38] Naeef AF, Mohammed AH, Mubarak AN (2020). Effects of cinnamon (*Cinnamomum cassia*) consumption on serum lipid profiles in Albino rats. J Lipids.

[39] Joohee O, Hyun-Sook K (2021). Anti-obese effect of cinnamon extracts dietary supplementation on serum lipids and body weight gain in high-fat-iet Induced obese mice model. Curr Dev Nutr.

[40] Zeynep T, Cemal O, Nurhan S, Vijaya J, Kazim S (2017). Cinnamon polyphenol extract inhibits hyperlipidemia and inflammation by modulation of transcription factors in high-fat diet-fed rats. Oxid Med Cell Longev.

[41] Pulungan A, Pane YS (2020). The benefit of cinnamon (Cinnamomum burmannii) in lowering total cholesterol levels after consumption of high-fat containing foods in white mice (Mus musculus) models. F1000Res..

[42] Abdelgadir AA, Hassan HM, Eltaher AM, Mohammed KG, Mohammed LA, Hago TB (2020). Hypolipidemic effect of Cinnamon (*Cinnamomum zeylanicum*) bark ethanolic extract on Triton X-100 induced hyperlipidemia in Albino rats. Med Aromat Plants.

[43] Mendis AWPK, Galbada ASP, Daya RW (2017). Bark extracts of Ceylon Cinnamon possess antilipidemic activities and bind bile acids in vitro. Evid Based Complement Alternat Med: eCAM.

[44] Verma S, Angadi S, Patil V, Mokashi A, Mathad J, Mummigatti U (2012). Growth, yield and quality of chrysanthemum (*Chrysanthemum morifolium Ramat*) Cv. Raja as influenced by integrated nutrient management. Karnataka. J Agric Sci.

[45] Yuan H, Jiang S, Liu Y, Daniyal M, Jian Y, Peng C, et al. The flower head of Chrysanthemum morifolium Ramat. (Juhua): A paradigm of flowers serving as Chinese dietary herbal medicine. J Ethnopharmacol. 2020;261:113043. 10.1016/j.jep.2020.113043 32593689

[46] Lin LZ, Harnly JM (2010). Identification of the phenolic components of chrysanthemum flower (*Chrysanthemum morifolium Ramat*). Food Chem.

[47] Gong J, Chu B, Gong L, Fang Z, Zhang X, Qiu S (2019). Comparison of phenolic compounds and the antioxidant activities of fifteen *Chrysanthemum morifolium Ramat* cv. ‘Hangbaiju’ in China. Antioxidants.

[48] Chen S, Liu J, Dong G, Zhang X, Liu Y, Sun W (2021). Flavonoids and caffeoylquinic acids in *Chrysanthemum morifolium* Ramat flowers: a potentially rich source of bioactive compounds. Food Chem.

[49] Yang L, Nuerbiye A, Cheng P, Wang JH, Li H (2017). Analysis of floral volatile components and antioxidant activity of different varieties of *Chrysanthemum morifolium*. Molecules.

[50] Lee Y, Lee J, Lee MS, Chang E, Kim Y (2021). Chrysanthemum morifolium flower extract ameliorates obesity-induced inflammation and increases the muscle mitochondria content and AMPK/SIRT1 activities in obese rats. Nutrients.

[51] Ma H, Liu S, Qu W, Huang Q, Li L, Chu F (2021). Comparison of the antioxidant activities of nonfumigated and sulphur-fumigated *Chrysanthemum morifolium* cv. Hang-ju induced by oxidative stress. Pharm Biol.

[52] Yang PF, Yang YN, He CY, Chen ZF, Yuan QS, Zhao SC (2019). New caffeoylquinic acid derivatives and flavanone glycoside from the flowers of *Chrysanthemum morifolium* and their bioactivities. Molecules.

[53] Sun J, Wang Z, Chen L, Sun G (2021). Hypolipidemic effects and preliminary mechanism of Chrysanthemum flavonoids, its main components luteolin and luteoloside in hyperlipidemia rats. Antioxidants (Basel).

[54] Mannucci C, Navarra M, Calapai F, Squeri R, Gangemi S, Calapai G (2017). Clinical pharmacology of citrus bergamia: a systematic review. Phytother Res.

[55] Nauman MC, Johnson JJ (2019). Clinical application of bergamot (*Citrus bergamia*) for reducing high cholesterol and cardiovascular disease markers. Integr Food Nutr Metab.

[56] Luo SM, Wu MH, Zhou Y, Huang ZH, Zhang Y, Ma ZG (2020). Herbalogical study on original plant and medicinal and edible values of *Citri Sarcodactylis Fructus*. Chin J Chin Mater Med.

[57] Zhao Y, Hu H, Peng T, Deng F, Xiang B, Kuang Y (2018). Research progress on chemical components, pharmacological action, development and application of Bergamot. Lishizhen Med Mater Med Res.

[58] Gabriele M, Frassinetti S, Caltavuturo L, Montero L, Dinelli G, Longo V (2017). Citrus bergamia powder: Antioxidant, antimicrobial and anti-inflammatory properties. J Funct Foods.

[59] Mollace V, Sacco I, Janda E, Malara C, Ventrice D, Colica C (2011). Hypolipemic and hypoglycaemic activity of bergamot polyphenols: from animal models to human studies. Fitoterapia.

[60] Ballistreri G, Amenta M, Fabroni S, Consoli V, Grosso S, Vanella L (2021). Evaluation of lipid and cholesterol-lowering effect of bioflavonoids from bergamot extract. Nat Prod Res.

[61] Huang Y, Tocmo R, Nauman MC, Haughan MA, Johnson JJ (2021). Defining the cholesterol lowering mechanism of Bergamot (*Citrus bergamia*) extract in HepG2 and Caco-2 Cells. Nutrients.

[62] Salerno R, Casale F, Calandruccio C, Procopio A (2016). Characterization of flavonoids in Citrus bergamia (Bergamot) polyphenolic fraction by liquid chromatography–high resolution mass spectrometry (LC/HRMS). PharmaNutrition.

[63] Musolino V, Gliozzi M, Scarano F, Bosco F, Scicchitano M, Nucera S (2020). Bergamot polyphenols improve dyslipidemia and pathophysiological features in a mouse model of non-alcoholic fatty liver disease. Sci Rep.

[64] Musolino V, Gliozzi M, Nucera S, Carresi C, Maiuolo J, Mollace R (2019). The effect of bergamot polyphenolic fraction on lipid transfer protein system and vascular oxidative stress in a rat model of hyperlipemia. Lipids Health Dis.

[65] Musolino V, Gliozzi M, Carresi C, Maiuolo J, Mollace R, Bosco F (2017). Lipid-lowering effect of bergamot polyphenolic fraction: role of pancreatic cholesterol ester hydrolase. J Biol Regul Homeost Agents.

[66] Sui GG, Xiao HB, Lu XY, Sun ZL (2018). Naringin activates AMPK resulting in altered expression of SREBPs, PCSK9, and LDLR to reduce body weight in obese C57BL/6J mice. J Agric Food Chem.

[67] Wu M, Liu L, Xing Y, Yang S, Li H, Cao Y (2020). Roles and mechanisms of Hawthorn and its extracts on atherosclerosis: A review. Front Pharmacol.

[68] Dong JQ, Chen JP, Gong SX, Xu J, Xu X, Zhang TJ (2021). Research progress on chemical constituents and pharmacological effects of Crataegi Fructus and predictive analysis on Q-Marker. Chin Tradit Herbal Drugs.

[69] He Z, Kwek E, Hao W, Zhu H, Liu J, Ma KY (2021). Hawthorn fruit extract reduced trimethylamine-N-oxide (TMAO)-exacerbated atherogenesis in mice via anti-inflammation and anti-oxidation. Nutr Metab (Lond).

[70] Orhan IE (2018). Phytochemical and pharmacological activity profile of *Crataegus oxyacantha* L. (Hawthorn) - a cardiotonic herb. Curr Med Chem.

[71] Hu C, Zhang Y, Liu G, Liu Y, Wang J, Sun B (2019). Untargeted metabolite profiling of adipose tissue in hyperlipidemia rats exposed to Hawthorn ethanol extracts. J Food Sci.

[72] Lee JJ, Lee HJ, Oh SW (2017). Antiobesity effects of Sansa (*Crataegi fructus*) on 3T3-L1 cells and on high-fat-high-cholesterol diet-induced obese rats. J Med Food.

[73] Qi J, Wang QZ, Yang J, Cai BM, Yu D (2020). Effects of *Crataegus cuneata Sieb*. et Zucc. and *Crataegus pinnatifida Bge*. Aqueous Extract on Human Endogenous Pancreatic Lipase. Food Nutr Chin..

[74] Zhong LX, Jiang ZY, Wang JN, Li XF, Xu LS (2019). Optimization of extraction technology of Hawthorn polysaccharides and its hypoglycemic and hypolipidemic activity. Sci Technol Food Ind..

[75] Ning SY, Teng D, Mu YN, Wang Y, Sun XY (2020). Study on the mechanism of hawthorn powder regulating blood lipid of high-fat diet mice based on intestinal microecology. Chin Tradit Pat Med.

[76] Bai BY, Zhou Q, Han X, Cai DW, Dong XH, Yan CJ (2017). Preliminary study on the antagonistic effect and its mechanism of hawthorn concentrated juice on hyperlipidemia mice. Food Sci Technol.

[77] Zhu R, Hou Y, Sun Y, Li T, Fan J, Chen G (2017). Pectin penta-oligogalacturonide suppresses intestinal bile acids absorption and downregulates the FXR-FGF15 axis in high-cholesterol fed mice. Lipids.

[78] Gao XJ, Luo SY, Tang KJ, Luo QS (2021). Effect of crude hawthorn glycoprotein on hypolipidemic and antioxidant activity in high-fat mice. Food Ferment Ind.

[79] Peng Y, Sun Q, Xu W, He Y, Jin W, Yuan L (2019). Vitexin ameliorates high fat diet-induced obesity in male C57BL/6J mice via the AMPKalpha-mediated pathway. Food Funct.

[80] Yang Y, Yao X, Li H (2021). Shanzha (*Fructus Crataegi*) attenuates atherosclerosis in high-fat diet-fed apolipoprotein e-knockout mice via regulating gut flora. Chin Arch Tradit Chin Med.

[81] Chen L, Li M, Yang Z, Tao W, Wang P, Tian X (2020). Gardenia jasminoides Ellis: ethnopharmacology, phytochemistry, and pharmacological and industrial applications of an important traditional Chinese medicine. J Ethnopharmacol.

[82] Toppo E, Darvin SS, Esakkimuthu S, Stalin A, Balakrishna K, Sivasankaran K (2017). Antihyperlipidemic and hepatoprotective effects of Gardenin A in cellular and high fat diet fed rodent models. Chem Biol Interact.

[83] Xiao W, Li S, Wang S, Ho CT (2017). Chemistry and bioactivity of *Gardenia jasminoides*. J Food Drug Anal.

[84] Lv S, Ding Y, Zhao H, Liu S, Zhang J, Wang J (2018). Therapeutic potential and effective components of the Chinese Herb Gardeniae Fructus in the treatment of senile disease. Aging Dis.

[85] Zhou YX, Zhang RQ, Rahman K, Cao ZX, Zhang H, Peng C (2019). Diverse pharmacological activities and potential medicinal benefits of Geniposide. Evid Based Complement Alternat Med.

[86] Chang R, Liu J, Luo Y, Huang T, Li Q, Wen J (2020). Isoflavones' effects on pharmacokinetic profiles of main iridoids from Gardeniae Fructus in rats. J Pharm Anal.

[87] Tang Z, Li L, Xia Z (2022). Exploring anti-nonalcoholic fatty liver disease mechanism of Gardeniae Fructus by network pharmacology, molecular docking, and experiment validation. ACS Omega.

[88] Shen B, Feng H, Cheng J, Li Z, Jin M, Zhao L (2020). Geniposide alleviates non-alcohol fatty liver disease via regulating Nrf2/AMPK/mTOR signalling pathways. J Cell Mol Med.

[89] Liu J, Li Y, Sun C, Liu S, Yan Y, Pan H (2020). Geniposide reduces cholesterol accumulation and increases its excretion by regulating the FXR-mediated liver-gut crosstalk of bile acids. Pharmacol Res.

[90] Cheng S, Zhou F, Xu Y, Liu X, Zhang Y, Gu M (2019). Geniposide regulates the miR-101/MKP-1/p38 pathway and alleviates atherosclerosis inflammatory injury in ApoE(-/-) mice. Immunobiology.

[91] Shen D, Zhao D, Yang X, Zhang J, He H, Yu C (2019). Geniposide against atherosclerosis by inhibiting the formation of foam cell and lowering reverse lipid transport via p38/MAPK signaling pathways. Eur J Pharmacol.

[92] Li N, Li L, Wu H, Zhou H (2019). Antioxidative property and molecular mechanisms underlying Geniposide-mediated therapeutic effects in diabetes mellitus and cardiovascular disease. Oxid Med Cell Longev.

[93] Guan L, Gong D, Yang S, Shen N, Zhang S, Li Y (2018). Genipin ameliorates diet-induced obesity via promoting lipid mobilization and browning of white adipose tissue in rats. Phytother Res.

[94] Zhong H, Chen K, Feng M, Shao W, Wu J, Chen K (2018). Genipin alleviates high-fat diet-induced hyperlipidemia and hepatic lipid accumulation in mice via miR-142a-5p/SREBP-1c axis. FEBS J.

[95] Ma X, Yang W, Kallio H, Yang B (2022). Health promoting properties and sensory characteristics of phytochemicals in berries and leaves of sea buckthorn (*Hippophaë rhamnoides*). Crit Rev Food Sci Nutr.

[96] Du W, Xiong CW, Ding J, Nybom H, Ruan CJ, Guo H (2019). Tandem mass tag based quantitative proteomics of developing sea buckthorn berries reveals candidate proteins related to lipid metabolism. J Proteome Res.

[97] Wang K, Xu Z, Liao X (2022). Bioactive compounds, health benefits and functional food products of sea buckthorn: a review. Crit Rev Food Sci Nutr..

[98] Micek A, Godos J, Del Rio D, Galvano F, Grosso G (2021). Dietary flavonoids and cardiovascular disease: a comprehensive dose-response meta-analysis. Mol Nutr Food Res.

[99] Zhang D, Wu G (2019). Research progress on chemical components and pharmacological action of seabuckthorn flavonoids. China Pharm.

[100] Sun C, Feng Y, Xie P, Song Z, Tang Z (2018). Hypolipidemic and hypoglycemic effects of total flavonoids from pomace of Fructus Hippophae. World Chin Med.

[101] Yang X, Wang Q, Pang ZR, Pan MR, Zhang W (2017). Flavonoid-enriched extract from Hippophae rhamnoides seed reduces high fat diet induced obesity, hypertriglyceridemia, and hepatic triglyceride accumulation in C57BL/6 mice. Pharm Biol.

[102] Xiao PT, Liu SY, Kuang YJ, Jiang ZM, Lin Y, Xie ZS (2021). Network pharmacology analysis and experimental validation to explore the mechanism of sea buckthorn flavonoids on hyperlipidemia. J Ethnopharmacol.

[103] Tie F, Ding J, Hu N, Dong Q, Chen Z, Wang H (2021). Kaempferol and kaempferide attenuate oleic acid-induced lipid accumulation and oxidative stress in HepG2 cells. Int J Mol Sci.

[104] Xue YT, Zhang XF, Zhang YH, Zhang BY, Zhang DJ (2019). Evaluation of the effect of seabuckthorn sterol on blood lipids. China Food Addit.

[105] Khan MA, Rahman AA, Islam S, Khandokhar P, Parvin S, Islam MB (2013). A comparative study on the antioxidant activity of methanolic extracts from different parts of Morus alba L (Moraceae). BMC Res Notes..

[106] Bhattacharjya D, Sadat A, Dam P, Buccini DF, Mondal R, Biswas T (2021). Current concepts and prospects of mulberry fruits for nutraceutical and medicinal benefits. Curr Opin Food Sci.

[107] Zhang H, Ma ZF, Luo X, Li X (2018). Effects of mulberry fruit (*Morus alba* L.) consumption on health outcomes: a mini-review. Antioxidants (Basel).

[108] Yuan Q, Zhao L (2017). The mulberry (*Morus alba* L.) fruit-a review of characteristic components and health benefits. J Agric Food Chem.

[109] Guo S, Bai L, Ho C-T, Bai N (2018). Characteristic components, biological activities and future prospective of Fructus Mori: a review. Curr Pharmacol Rep.

[110] Lee S, Lee MS, Chang E, Lee Y, Lee J, Kim J (2020). Mulberry Fruit Extract Promotes Serum HDL-cholesterol levels and suppresses hepatic microRNA-33 expression in rats fed high cholesterol/cholic acid diet. Nutrients.

[111] Lee MS, Kim Y (2020). Mulberry fruit extract ameliorates adipogenesis via increasing AMPK activity and downregulating microRNA-21/143 in 3T3-L1 adipocytes. J Med Food.

[112] Suriya C, Usana C, Rachanee C, Watcharakorn D, Kittiwoot T-o, Supaporn P (2021). Dried mulberry fruit ameliorates cardiovascular and liver histopathological changes in high-fat diet-induced hyperlipidemic mice. J Tradit Complement Med.

[113] Chen C, You LJ, Huang Q, Fu X, Zhang B, Liu RH (2018). Modulation of gut microbiota by mulberry fruit polysaccharide treatment of obese diabetic db/db mice. Food Funct.

[114] Jiao Y, Wang X, Jiang X, Kong F, Wang S, Yan C (2017). Antidiabetic effects of Morus alba fruit polysaccharides on high-fat diet- and streptozotocin-induced type 2 diabetes in rats. J Ethnopharmacol.

[115] Zhang R, Zhang Q, Zhu S, Liu B, Liu F, Xu Y (2022). Mulberry leaf (*Morus alba* L): a review of its potential influences in mechanisms of action on metabolic diseases. Pharmacol Res.

[116] Peng CH, Lin HT, Chung DJ, Huang CN, Wang CJ (2018). Mulberry leaf extracts prevent obesity-induced NAFLD with regulating adipocytokines, inflammation and oxidative stress. J Food Drug Anal.

[117] Zhong Y, Wu S, Chen F, He M, Lin J (2019). Isolation of high gamma-aminobutyric acid-producing lactic acid bacteria and fermentation in mulberry leaf powders. Exp Ther Med.

[118] Ann JY, Eo H, Lim Y (2015). Mulberry leaves (*Morus alba* L.) ameliorate obesity-induced hepatic lipogenesis, fibrosis, and oxidative stress in high-fat diet-fed mice. Genes Nutr.

[119] He L, Zhou W, Wang C, Yang F, Chen X, Zhang Q (2019). Effect of cellulase and Lactobacillus casei on ensiling characteristics, chemical composition, antioxidant activity, and digestibility of mulberry leaf silage. J Dairy Sci.

[120] Sheng Y, Zheng S, Ma T, Zhang C, Ou X, He X (2017). Mulberry leaf alleviates streptozotocin-induced diabetic rats by attenuating NEFA signaling and modulating intestinal microflora. Sci Rep.

[121] Huang J, Wang Y, Ying C, Liu L, Lou Z (2018). Effects of mulberry leaf on experimental hyperlipidemia rats induced by high-fat diet. Exp Ther Med.

[122] Lee E, Lee MS, Chang E, Kim CT, Choi AJ, Kim IH (2021). High hydrostatic pressure extract of mulberry leaves ameliorates hypercholesterolemia via modulating hepatic microRNA-33 expression and AMPK activity in high cholesterol diet fed rats. Food Nutr Res.

[123] Lee Y, Lee E, Lee M-S, Lee S, Kim C, Kim Y (2019). Hypolipidemic effect of mulberry leaf extract in rats fed a high-cholesterol diet (P06–014-19). Current developments in nutrition.

[124] Meng Q, Qi X, Fu Y, Chen Q, Cheng P, Yu X (2020). Flavonoids extracted from mulberry (*Morus alba* L.) leaf improve skeletal muscle mitochondrial function by activating AMPK in type 2 diabetes. J Ethnopharmacol.

[125] Hu Y, Xu J, Chen Q, Liu M, Wang S, Yu H (2020). Regulation effects of total flavonoids in *Morus alba* L. on hepatic cholesterol disorders in orotic acid induced NAFLD rats. BMC Complement Med Ther.

[126] Liao S, Long X, Zou Y, Liu F, Li Q (2021). Mulberry leaf phenolics and fiber exert anti-obesity through the gut microbiota-host metabolism pathway. J Food Sci.

[127] Lee YJ, Hsu JD, Lin WL, Kao SH, Wang CJ (2017). Upregulation of caveolin-1 by mulberry leaf extract and its major components, chlorogenic acid derivatives, attenuates alcoholic steatohepatitis via inhibition of oxidative stress. Food Funct.

[128] Li R, Xue Z, Jia Y, Wang Y, Li S, Zhou J (2021). Polysaccharides from mulberry (*Morus alba* L.) leaf prevents obesity by inhibiting pancreatic lipase in high-fat diet induced mice. Int J Biol Macromol.

[129] Ye LH, Kong LT, Yan MZ, Cao FR, Wang LS, Liao YH (2016). Lotus leaf alkaloid fraction can strongly inhibit CYP2D6 isoenzyme activity. J Ethnopharmacol.

[130] Li M, Zhao Z, Xuan J, Li Z, Ma T (2020). Advances in studies on chemical constituents and pharmacological effects of lotus leaves. J Liaoning Univ Tradit Chin Med.

[131] Lou ZH, Cheng B, Xia BH, Wang YP, Xu H, Zhang GJ (2017). Effects of Folium Nelumbinis on experimental nonalcoholic fatty liver disease induced by high glucose and high fat diet. Chin J Chin Mater Med.

[132] Wan Y, Xia J, Xu JF, Chen L, Yang Y, Wu JJ (2022). Nuciferine, an active ingredient derived from lotus leaf, lights up the way for the potential treatment of obesity and obesity-related diseases. Pharmacol Res.

[133] Cui H, Li Y, Cao M, Liao J, Liu X, Miao J (2020). Untargeted metabolomic analysis of the effects and mechanism of nuciferine treatment on rats with nonalcoholic fatty liver disease. Front Pharmacol.

[134] He B, Gao Y, Sun H, Wang J (2020). Effects of nuciferine on non alcoholic fatty liver disease and the relative mechanisms based on SREBP signaling pathway. J Tianjin Univ Tradit Chin Med.

[135] Yu Y, Lu J, Sun L, Lyu X, Chang XY, Mi X (2021). *Akkermansia muciniphila*: a potential novel mechanism of nuciferine to improve hyperlipidemia. Biomed Pharmacother.

[136] Shi Z, Fang ZY, Gao XX, Yu H, Zhu YW, Ouyang HL (2021). Nuciferine improves high-fat diet-induced obesity via reducing intestinal permeability by increasing autophagy and remodeling the gut microbiota. Food Funct.

[137] Wang Y, Yao W, Li B, Qian S, Wei B, Gong S (2020). Nuciferine modulates the gut microbiota and prevents obesity in high-fat diet-fed rats. Exp Mol Med.

[138] Xu H, Wang L, Yan K, Zhu H, Pan H, Yang H (2021). Nuciferine inhibited the differentiation and lipid accumulation of 3T3-L1 preadipocytes by regulating the expression of lipogenic genes and adipokines. Front Pharmacol.

[139] Ding C, Yin P, Zhao Q, Su L (2020). Nuciferine promotes autophagy and reduces macrophage foaming by inhibiting PI3K/Akt/mTOR signaling pathway. Chin J Pathophysiol.

[140] Zou J, Zhao Z, Wu J, Wang G, Tang C (2018). Nuciferine promotes ABCA1 expression and cholesterol efflux in THP-1 macrophage-derived foam cells and its mechanism. Chin J Arterioscler.

[141] Li H, Mei Q, Zhao Z, Yang D, Song Y, Zheng Y (2019). Overview of studies on chemical constituents, pharmacological action and comprehensive utilization of Citri Reticulatae Pericarpium. Lishizhen Med Mater Med Res.

[142] Cheng H, Wu X, Ni G, Wang S, Peng W, Zhang H (2020). Citri Reticulatae Pericarpium protects against isoproterenol-induced chronic heart failure via activation of PPARgamma. Ann Transl Med.

[143] Yu X, Sun S, Guo Y, Liu Y, Yang D, Li G (2018). Citri Reticulatae Pericarpium (Chenpi): Botany, ethnopharmacology, phytochemistry, and pharmacology of a frequently used traditional Chinese medicine. J Ethnopharmacol.

[144] Park JS, Cho EY, Kim YS, Kwon E, Han KM, Ku SY (2020). In vivo and in vitro safety evaluation of fermented Citrus sunki peel extract: acute and 90-day repeated oral toxicity studies with genotoxicity assessment. BMC Complement Med Ther.

[145] Zheng GD, Zhou P, Yang H, Li YS, Li P, Liu EH (2013). Rapid resolution liquid chromatography-electrospray ionisation tandem mass spectrometry method for identification of chemical constituents in Citri Reticulatae Pericarpium. Food Chem.

[146] Yu JJ, Su J, Yan MQ, Lou ZH, Lyu GY (2019). Correlation between lipid-lowering efficacy and components of Pericarpium Citri Reticulatae. Chin J Chin Mater Med.

[147] Lu XY, Shi XJ, Hu A, Wang JQ, Ding Y, Jiang W (2020). Feeding induces cholesterol biosynthesis via the mTORC1-USP20-HMGCR axis. Nature.

[148] Yu JJ, Du YZ, Su J, Yan MQ, Ji WN, Wu YL (2021). Preventive effect and mechanism of Citri Reticulatae Pericarpium on hypercholesterolemia rats. Chin Tradit Pat Med.

[149] Du YZ, Su J, Yan MQ, Chen SH, Lyu GY, Yu JJ (2021). Improvement effect and mechanism of ethanol extract from Citri Reticulatae Pericarpium on triglyceride in hyperlipidemia model rat. Chin J Chin Mater Med.

[150] Li A, Wang N, Li N, Li B, Yan F, Song Y (2021). Modulation effect of chenpi extract on gut microbiota in high-fat diet-induced obese C57BL/6 mice. J Food Biochem.

[151] Okagu IU, Ndefo JC, Aham EC, Udenigwe CC (2021). Zanthoxylum Species: a review of traditional uses, phytochemistry and pharmacology in relation to cancer, infectious diseases and sickle cell anemia. Front Pharmacol.

[152] Xiang L, Liu Y, Xie C, Li X, Yu Y, Ye M (2016). The chemical and genetic characteristics of Szechuan pepper (Zanthoxylum bungeanum and Z. armatum) cultivars and their suitable habitat. Front. Plant Sci..

[153] Bautista DM, Sigal YM, Milstein AD, Garrison JL, Zorn JA, Tsuruda PR (2008). Pungent agents from Szechuan peppers excite sensory neurons by inhibiting two-pore potassium channels. Nat Neurosci.

[154] Wagner H, Bauer R, Melchart D, Xiao PG, Staudinger A (2011). Pericarpium Zanthoxyli *Huajiao*. Chromatographic fingerprint analysis of herbal medicines.

[155] Zhang M, Wang J, Zhu L, Li T, Jiang W, Zhou J (2017). *Zanthoxylum Bungeanum* maxim (Rutaceae): a systematic review of its traditional uses, botany, phytochemistry, pharmacology, pharmacokinetics, and toxicology. Int J Mol Sci.

[156] Ombito JO (2021). Phytochemistry and pharmacology of the genus *Zanthoxylum* (Rutaceae): a review. Nat Prod J.

[157] Wang L, Fan W, Zhang M, Zhang Q, Li L, Wang J (2019). Antiobesity, regulation of lipid metabolism, and attenuation of liver oxidative stress effects of hydroxy-alpha-sanshool isolated from Zanthoxylum bungeanum on high-fat diet-induced hyperlipidemic rats. Oxid Med Cell Longev.

[158] Obidiegwu JE, Lyons JB, Chilaka CA (2020). The Dioscorea genus (yam)—an appraisal of nutritional and therapeutic potentials. Foods.

[159] Okwu D, Ndu C (2006). Evaluation of the phytonutrients, mineral and vitamin contents of some varieties of yam (*Dioscorea* sp.). Int J Mol Med Adv Sci.

[160] Zhang L, Ng TB, Lam JKW, Wang SW, Lao L, Zhang KY (2019). Research and development of proteins and peptides with therapeutic potential from yam tubers. Curr Protein Pept Sci.

[161] Jesus M, Martins AP, Gallardo E, Silvestre S (2016). Diosgenin: Recent highlights on pharmacology and analytical methodology. J Anal Methods Chem.

[162] Kusano Y, Tsujihara N, Masui H, Shibata T, Uchida K, Takeuchi W (2019). Diosgenin supplementation prevents lipid accumulation and induces skeletal muscle-fiber hypertrophy in rats. J Nutr Sci Vitaminol (Tokyo).

[163] Meenu M, Xu B (2019). A critical review on anti-diabetic and anti-obesity effects of dietary resistant starch. Crit Rev Food Sci Nutr.

[164] Li T, Teng H, An F, Huang Q, Chen L, Song H (2019). The beneficial effects of purple yam (*Dioscorea alata* L.) resistant starch on hyperlipidemia in high-fat-fed hamsters. Food Funct.

[165] Yang XX, Wang X, Shi TT, Dong JC, Li FJ, Zeng LX (2019). Mitochondrial dysfunction in high-fat diet-induced nonalcoholic fatty liver disease: the alleviating effect and its mechanism of *Polygonatum kingianum*. Biomed Pharmacother.

[166] Zhao P, Zhao C, Li X, Gao Q, Huang L, Xiao P (2018). The genus *Polygonatum*: a review of ethnopharmacology, phytochemistry and pharmacology. J Ethnopharmacol.

[167] Cui X, Wang S, Cao H, Guo H, Li Y, Xu F (2018). A review: The bioactivities and pharmacological applications of *Polygonatum sibiricum* polysaccharides. Molecules.

[168] Sun T, Zhang H, Li Y, Liu Y, Dai W, Fang J (2020). Physicochemical properties and immunological activities of polysaccharides from both crude and wine-processed *Polygonatum sibiricum*. Int J Biol Macromol.

[169] Yelithao K, Surayot U, Park W, Lee S, Lee DH, You S (2019). Effect of sulfation and partial hydrolysis of polysaccharides from *Polygonatum sibiricum* on immune-enhancement. Int J Biol Macromol.

[170] Yang XX, Wei JD, Mu JK, Liu X, Dong JC, Zeng LX (2018). Integrated metabolomic profiling for analysis of antilipidemic effects of *Polygonatum kingianum* extract on dyslipidemia in rats. World J Gastroenterol.

[171] Kong X, Liu JJ, Li H, Chen ZB (2018). Effect of polysaccharides from *Polygonatum sibiricum* on lipid-metabolism related mRNA and protein expression in hyperlipidemic mice. Chin J Chin Mater Med.

[172] Gu W, Wang Y, Zeng L, Dong J, Bi Q, Yang X (2020). Polysaccharides from *Polygonatum kingianum* improve glucose and lipid metabolism in rats fed a high fat diet. Biomed Pharmacother.

[173] Chai Y, Luo J, Bao Y (2021). Effects of *Polygonatum sibiricum* saponin on hyperglycemia, gut microbiota composition and metabolic profiles in type 2 diabetes mice. Biomed Pharmacother.

[174] Zhai L, Wang X (2018). SyringaresinoldiObetaDglucoside, a phenolic compound from *Polygonatum sibiricum*, exhibits an antidiabetic and antioxidative effect on a streptozotocininduced mouse model of diabetes. Mol Med Rep.

[175] Shahrajabian MH (2019). A review of Astragalus species as foodstuffs, dietary supplements, a traditional Chinese medicine and a part of modern pharmaceutical science. Appl Ecol Env Res.

[176] Zhang LJ, Liu HK, Hsiao PC, Kuo LM, Lee IJ, Wu TS (2011). New isoflavonoid glycosides and related constituents from astragali radix (*Astragalus membranaceus*) and their inhibitory activity on nitric oxide production. J Agric Food Chem.

[177] Gong AGW, Duan R, Wang HY, Kong XP, Dong TTX, Tsim KWK (2018). Evaluation of the pharmaceutical properties and value of Astragali Radix. Medicines (Basel).

[178] Song JZ, Yiu HH, Qiao CF, Han QB, Xu HX (2008). Chemical comparison and classification of *Radix Astragali* by determination of isoflavonoids and astragalosides. J Pharm Biomed Anal.

[179] Fu J, Wang Z, Huang L, Zheng S, Wang D, Chen S (2014). Review of the botanical characteristics, phytochemistry, and pharmacology of *Astragalus membranaceus* (Huangqi). Phytother Res.

[180] Zhang CH, Yang X, Wei JR, Chen NM, Xu JP, Bi YQ (2021). Ethnopharmacology, phytochemistry, pharmacology, toxicology and clinical applications of *Radix Astragali*. Chin J Integr Med.

[181] Su HF, Shaker S, Kuang Y, Zhang M, Ye M, Qiao X (2021). Phytochemistry and cardiovascular protective effects of Huang-Qi (*Astragali Radix*). Med Res Rev.

[182] Wang Z, Li XL, Hong KF, Zhao TT, Dong RX, Wang WM (2021). Total flavonoids of Astragalus ameliorated bile acid metabolism dysfunction in diabetes mellitus. Evid Based Complement Alternat Med.

[183] Ma C, Zhang J, Yang S, Hua Y, Su J, Shang Y (2020). Astragalus flavone ameliorates atherosclerosis and hepatic steatosis via inhibiting lipid-disorder and inflammation in ApoE(-/-) mice. Front Pharmacol.

[184] Qian W, Qian Q, Cai X, Han R, Yang W, Zhang X (2019). Astragaloside IV inhibits oxidized lowdensity lipoproteininduced endothelial damage via upregulation of miR1403p. Int J Mol Med.

[185] Zhou B, Zhou DL, Wei XH, Zhong RY, Xu J, Sun L (2017). Astragaloside IV attenuates free fatty acid-induced ER stress and lipid accumulation in hepatocytes via AMPK activation. Acta Pharmacol Sin.

[186] Wang Z, Zhu Y, Zhang Y, Zhang J, Ji T, Li W (2020). Protective effects of AS-IV on diabetic cardiomyopathy by improving myocardial lipid metabolism in rat models of T2DM. Biomed Pharmacother.

[187] Luo D, Dong X, Huang J, Huang C, Fang G, Huang Y (2021). Pueraria lobata root polysaccharide alleviates glucose and lipid metabolic dysfunction in diabetic db/db mice. Pharm Biol.

[188] Zhang Z, Lam TN, Zuo Z (2013). Radix Puerariae: an overview of its chemistry, pharmacology, pharmacokinetics, and clinical use. J Clin Pharmacol.

[189] Wong KH, Razmovski-Naumovski V, Li KM, Li GQ, Chan K (2015). Comparing morphological, chemical and anti-diabetic characteristics of *Puerariae Lobatae Radix* and *Puerariae Thomsonii Radix*. J Ethnopharmacol.

[190] Buhlmann E, Horvath C, Houriet J, Kiehlmann E, Radtke J, Marcourt L (2019). Puerariae lobatae root extracts and the regulation of brown fat activity. Phytomedicine.

[191] Liu YS, Yuan MH, Zhang CY, Liu HM, Liu JR, Wei AL (2021). Puerariae Lobatae radix flavonoids and puerarin alleviate alcoholic liver injury in zebrafish by regulating alcohol and lipid metabolism. Biomed Pharmacother.

[192] Yuan G, Shi S, Jia Q, Shi J, Shi S, Zhang X (2021). Use of network pharmacology to explore the mechanism of Gegen (*Puerariae lobatae Radix*) in the treatment of type 2 diabetes mellitus associated with hyperlipidemia. Evid Based Complement Alternat Med.

[193] Jung HW, Kang AN, Kang SY, Park YK, Song MY (2017). The root extract of Pueraria lobata and its main compound, puerarin, prevent obesity by increasing the energy metabolism in skeletal muscle. Nutrients.

[194] Xu DX, Guo XX, Zeng Z, Wang Y, Qiu J (2021). Puerarin improves hepatic glucose and lipid homeostasis in vitro and in vivo by regulating the AMPK pathway. Food Funct.

[195] Hou B, Zhao Y, Qiang G, Yang X, Xu C, Chen X (2018). Puerarin mitigates diabetic hepatic steatosis and fibrosis by inhibiting TGF-beta signaling pathway activation in type 2 diabetic rats. Oxid Med Cell Longev.

[196] Rao Y, Wen Q, Liu R, He M, Jiang Z, Qian K (2020). PL-S2, a homogeneous polysaccharide from *Radix Puerariae lobatae*, attenuates hyperlipidemia via farnesoid X receptor (FXR) pathway-modulated bile acid metabolism. Int J Biol Macromol.

[197] Ju MS, Kim HG, Choi JG, Ryu JH, Hur J, Kim YJ (2010). *Cassiae* Semen, a seed of *Cassia* obtusifolia, has neuroprotective effects in Parkinson's disease models. Food Chem Toxicol.

[198] Dong YJ, Jiang YQ, Liu Y, Chen JP, Gai XH, Tian CW (2021). Research progress on chemical composition and pharmacological effects of *Cassiae* Semen and predictive analysis on quality markers. Chin Tradit Herbal Drugs.

[199] Yu F, Sun L, Xu L, Xiao P, Miao J (2018). Research progress on modern application of *Cassiae* Semen. Mod Chin Med.

[200] Dong X, Fu J, Yin X, Yang C, Zhang X, Wang W (2017). *Cassiae* Semen: a review of its phytochemistry and pharmacology (Review). Mol Med Rep.

[201] Zhu Z, Zhang S, Zheng Y (2021). Effect of extracts of *Cassiae* Semen on lipid, liver and kidney function in hyperlipidemia rats. Prev Med.

[202] Qi ZL, Bian Y, Cai HQ, Li X, Zhang Y (2018). Effects of Semen *Cassiae* extract to blood lipid level of hyperlipidemia rats. J Harbin Med Univ.

[203] Luo H, Wu H, Wang L, Xiao S, Lu Y, Liu C (2021). Hepatoprotective effects of *Cassiae* Semen on mice with non-alcoholic fatty liver disease based on gut microbiota. Commun Biol.

[204] Meng Y, Liu Y, Fang N, Guo Y (2019). Hepatoprotective effects of *Cassia* Semen ethanol extract on non-alcoholic fatty liver disease in experimental rat. Pharm Biol.

[205] Xu P, Sun X, Huang Y, Shan Y, Wu Y (2018). Research progress on lipid lowering effective components of semen *Cassiae*. Chin Arch Tradit Chin Med.

[206] Li Y, Hou W, Wu J, Song B, Chen W (2019). The affection of cassia glycosides on SREBP-1c and PPARα in liver of nonalcoholic fatty liver disease rats. Med J West Chin.

[207] Ma J, Liu X, Yu J, Sun J (2021). Effect of 1, 8-Dihydroxyanthraquinone on the imbalance of lipid metabolism via regulation of expression of CYP7A1 and 3-hydroxy-3-methylglutaryl coenzyme a reductase mRNA in hyperlipidemic mice. Pharmacogn Mag.

[208] Vadivel V, Doss A, Pugalenthi M (2010). Evaluation of nutritional value and protein quality of raw and differentially processed sword bean [*Canavalia gladiata* (Jacq.) DC.] seeds. Afr J Food, Agric, Nutr Dev.

[209] An HJ, Kim EH, Lee HJ, Cho JY, Moon JH (2020). New caryophyllene-type sesquiterpene and flavonol tetraglycoside with sixteen known compounds from sword bean (*Canavalia gladiata*). Food Sci Biotechnol.

[210] Hwang KA, Heo W, Hwang HJ, Han BK, Song MC, Kim YJ (2020). Anti-inflammatory effect of immature sword bean pod (*Canavalia gladiata*) in lipopolysaccharide-induced RAW264. 7 cells. J Med Food.

[211] Ghaben AL, Scherer PE (2019). Adipogenesis and metabolic health. Nat Rev Mol Cell Biol.

[212] Yujeong C, DaSom K, MinChul L, Seulgi P, JooWon L, AeSon O (2021). Effects of bacillus subtilis-fermented white sword bean extract on adipogenesis and lipolysis of 3T3-L1 adipocytes. Foods.

[213] Anitha K, Mohana Lakshmi S, Satyanarayana SV (2020). Antidiabetic, lipid lowering and antioxidant potentiating effect of *Canavalia* species in high fat diet-streptozotocin induced model. Adv Tradit Med.

[214] Sridhar KR, Sharma BB, Murthy HN, Paek KY (2020). Bioactive compounds of Jack beans (*Canavalia* species). Bioactive compounds in underutilized vegetables and legumes.

[215] Naufalina MD, Sofro MA, Anjani G (2018). *Canavalia* ensiformis protein extract effect toward serum lipid profile of hypercholesterolemic Sprague Dawley rat. Jurnal Kesehatan Masyarakat.

[216] Im AR, Kim YH, Lee HW, Song KH (2016). Water extract of Dolichos lablab attenuates hepatic lipid accumulation in a cellular nonalcoholic fatty liver disease model. J Med Food.

[217] Chun E, Yoon S, Parveen A, Jin M (2018). Alleviation of irritable bowel syndrome-like symptoms and control of gut and brain responses with oral administration of Dolichos lablab L. in a mouse model. Nutrients.

[218] Al-Snafi AE (2017). The pharmacology and medical importance of Dolichos lablab (Lablab purpureus)-a review. IOSR J Pharm.

[219] Singh V, Kudesia R (2020). Review on taxonomical and pharmacological status of Dolichos lablab. Curr Trends Biotechnol Pharm.

[220] Suh DH, Lee HW, Jung ES, Singh D, Kim SH, Lee CH (2017). In vivo metabolomic interpretation of the anti-obesity effects of hyacinth bean (Dolichos lablab L.) administration in high-fat diet mice. Mol Nutr Food Res..

[221] Im AR, Kim YH, Kim YH, Yang WK, Kim SH, Song KH (2017). Dolichos lablab protects against nonalcoholic fatty liver disease in mice fed high-fat diets. J Med Food.

[222] Xi S, Qian L, Tong H, Yue L, Zhao H, Wang D (2013). Toxicity and clinical reasonable application of Taoren (Semen *Persicae*) based on ancient and modern literature research. J Tradit Chin Med.

[223] Zhang Y, Wei J, Lu C, He Z, Gan J, Feng X (2021). Chemical components and pharmacological action for *Persicae* Semen and predictive analysis on Q-marker. Chin Arch Tradit Chin Med.

[224] Zhao Y, Niu K, Tang D, Liang Q, Shu B, Li C (2015). Research on pharmacological effects of Peach Kernel. Liaoning J Tradit Chin Med.

[225] Lv J, Xiong W, Lei T, Wang H, Sun M, Hao E (2017). Amygdalin ameliorates the progression of atherosclerosis in LDL receptordeficient mice. Mol Med Rep.

[226] Wang Y, Jia Q, Zhang Y, Wei J, Liu P (2020). Amygdalin attenuates atherosclerosis and plays an anti-inflammatory role in ApoE knock-out mice and bone marrow-derived macrophages. Front Pharmacol.

[227] Pelentir N, Block JM, Monteiro Fritz AR, Reginatto V, Amante ER (2011). Production and chemical characterization of peach (Prunus persica) kernel flour. J Food Process Eng.

[228] Hao E, Pang G, Du Z, Lai YH, Chen JR, Xie J (2019). Peach kernel oil downregulates expression of tissue factor and reduces atherosclerosis in ApoE knockout mice. Int J Mol Sci.

[229] Iranshahy M, Javadi B, Iranshahi M, Jahanbakhsh SP, Mahyari S, Hassani FV (2017). A review of traditional uses, phytochemistry and pharmacology of Portulaca oleracea L. J Ethnopharmacol.

[230] Zheng G, Mo F, Ling C, Peng H, Gu W, Li M (2018). Portulaca oleracea L. alleviates liver injury in streptozotocin-induced diabetic mice. Drug Des Devel Ther.

[231] Chen D, Yao JN, Liu T, Zhang HY, Li RR, Zhang ZJ (2019). Research and application of Portulaca oleracea in pharmaceutical area. Chin Herb Med.

[232] Lee JH, Park JE, Han JS (2020). Portulaca oleracea L. extract reduces hyperglycemia via PI3k/Akt and AMPK pathways in the skeletal muscles of C57BL/Ksj-db/db mice. J Ethnopharmacol.

[233] Melilli MG, Pagliaro A, Scandurra S, Gentile C, Stefano VD (2020). Omega-3 rich foods: durum wheat spaghetti fortified with Portulaca oleracea. Food Biosci.

[234] Shahidi F, Ambigaipalan P (2018). Omega-3 polyunsaturated fatty acids and their health benefits. Annu Rev Food Sci Technol.

[235] Samir D, Kaouther A, Manal D (2017). Polysaccharides and ascorbic acid content and the effect of aqueous extract of Portulaca Oleracea in high-fat diet-induced obesity, dyslipidemia and liver damage in albino wistar rats. Alger J Arid Environ.

[236] Djellouli F, Krouf D, Lacaille-Dubois M, Bouchenak M (2018). Portulaca oleracea reduces lipemia, glycemia, and oxidative stress in streptozotocin-induced diabetic rats fed cholesterol-enriched diet. J Pharm Res Int..

[237] Dreny EGE (2020). Antidiabetic activity of aerial parts and seeds of Purslane (Portulaca oleracea) on diabetic rats. Eur J Nutr Food Saf.

[238] Yahiaoui Z, Sherazede B, Malika B (2017). Aqueous extract of Portulaca oleracea prevents lipid peroxidation and increases serum paraoxonase-1 activity, in rats fed cholesterol enriched-diet. Nutr Santé.

[239] Jung JH, Hwang SB, Park HJ, Jin GR, Lee BH (2021). Antiobesity and antidiabetic effects of Portulaca oleracea powder intake in high-fat diet-induced obese C57BL/6 mice. Evid Based Complement Alternat Med.

